# Inhibition of *Mycobacterium tuberculosis* dihydrodipicolinate synthase by alpha-ketopimelic acid and its other structural analogues

**DOI:** 10.1038/srep30827

**Published:** 2016-08-09

**Authors:** Priyanka Shrivastava, Vikas Navratna, Yumnam Silla, Rikeshwer P. Dewangan, Atreyi Pramanik, Sarika Chaudhary, GeethaVani Rayasam, Anuradha Kumar, Balasubramanian Gopal, Srinivasan Ramachandran

**Affiliations:** 1 Functional Genomics Unit, Council of Scientific and Industrial Research-Institute of Genomics and Integrative Biology (CSIR-IGIB), South Campus, New Delhi 110025, India; 2 Academy of Scientific and Innovative Research, CSIR-IGIB South Campus, New Delhi 110025, India; 3 Molecular Biophysics Unit, Indian Institute of Science, Bangalore, 560012, India; 4 Biotechnology Group (BIF center), Biological Science & Technology Division (BSTD), CSIR-North-East Institute of Science and Technology (CSIR-NEIST), Jorhat, Assam, 785006, India; 5 Open Source Drug Discovery Unit (OSDD), CSIR-IGIB, New Delhi, 110001, India

## Abstract

The *Mycobacterium tuberculosis* dihydrodipicolinate synthase (Mtb-*dapA*) is an essential gene. Mtb-DapA catalyzes the aldol condensation between pyruvate and L-aspartate-beta-semialdehyde (ASA) to yield dihydrodipicolinate. In this work we tested the inhibitory effects of structural analogues of pyruvate on recombinant Mtb-DapA (Mtb-rDapA) using a coupled assay with recombinant dihydrodipicolinate reductase (Mtb-rDapB). Alpha-ketopimelic acid (α-KPA) showed maximum inhibition of 88% and IC_50_ of 21 μM in the presence of pyruvate (500 μM) and ASA (400 μM). Competition experiments with pyruvate and ASA revealed competition of α-KPA with pyruvate. Liquid chromatography-mass spectrometry (LC-MS) data with multiple reaction monitoring (MRM) showed that the relative abundance peak of final product, 2,3,4,5-tetrahydrodipicolinate, was decreased by 50%. Thermal shift assays showed 1 °C Tm shift of Mtb-rDapA upon binding α-KPA. The 2.4 Å crystal structure of Mtb-rDapA-α-KPA complex showed the interaction of critical residues at the active site with α-KPA. Molecular dynamics simulations over 500 ns of pyruvate docked to Mtb-DapA and of α-KPA-bound Mtb-rDapA revealed formation of hydrogen bonds with pyruvate throughout in contrast to α-KPA. Molecular descriptors analysis showed that ligands with polar surface area of 91.7 Å^2^ are likely inhibitors. In summary, α-hydroxypimelic acid and other analogues could be explored further as inhibitors of Mtb-DapA.

Tuberculosis (TB) caused by *Mycobacterium tuberculosis* (Mtb) is a leading killer among infectious diseases worldwide. TB alone accounts for 1.5 million deaths per year^1^. The principal route of infection is through inhalation of aerosols containing Mtb bacteria thereby causing pulmonary TB. Mtb also colonizes other tissues of the body causing extra-pulmonary TB. Further, Mtb, including its multidrug and extensively drug-resistant strains, transforms into a persistent state and can remain for long times in the host^2^.

The cell wall of Mtb is particularly rigid, rich in mycolic acids covalently linked to the arabinogalactan layer and cross-linked by the peptidoglycan layer^3^. Therefore the enzymes of the cell wall biosynthesis and maintenance pathways constitute promising targets for the identification of new antitubercular therapeutics. The Mtb dihydrodipicolinate synthase (Mtb-DapA) is one such target^4^.

The synthesis of lysine and meso-diaminopimelate (M-DAP) in mycobacteria are accomplished through the diaminopimelate (DAP) pathway^5^. Both lysine and M-DAP are essential for bacterial growth and survival^6^. Lysine is needed for protein biosynthesis. M-DAP is an important component for crosslinks in the peptidoglycan layer, which has been implicated as a potential virulence factor^7^. Inhibitors of enzymes in the DAP pathway could be considered for development of new antibacterial drugs because this pathway is indispensable for bacteria and is absent in humans^5^.

The DAP pathway (Supplementary Figure S1) begins with phosphorylation of L-aspartate to L-β-aspartyl-4-phosphate catalyzed by aspartokinase. Next, L-β-aspartyl-4-phosphate is reduced to L-aspartate-beta-semialdehyde (ASA) catalyzed by aspartate semialdehyde dehydrogenase. This is followed by a Schiff base formation with pyruvate and addition of ASA to form (4S)-4-hydroxy-2,3,4,5-tetrahydro-(2S)-dipicolinate (HTPA) catalyzed by dihydrodipicolinate synthase (Mtb-DapA)^8^ (Supplementary Figure S2). Next, HTPA is reduced to 2,3,4,5-tetrahydrodipicolinic acid (THDP) catalyzed by dihydrodipicolinate reductase (Mtb-DapB) using NADPH^9^ as an electron donor. In mycobacteria, THDP undergoes a series of biochemical transformations to yield meso-diaminopimelate (M-DAP)^10^.

The crystal structures of Mtb-DapA^11^ and its homologues from *Escherichia coli*^12^, *Nicotiana sylvestris*^13^, *Thermotoga maritima*^14^, *Clostridium botulinum*^15^, *Corynebacterium glutamicum*^16^, *Bacillus anthracis*^17^ and *Staphylococcus aureus*^18^ have been determined. Mtb-DapA belongs to the subfamily of N-acetylneuraminate lyase (α/β)8 proteins. It contains an N-terminal (α/β)8 TIM barrel catalytic core and an α-helical C-terminal domain^19^. The active site of the enzyme is located at the center of the monomer towards the C-terminal end of the TIM barrel. The active site residues Thr54, Thr55, Tyr143, Arg148 and Lys171 of Mtb-DapA are largely conserved among its homologues^11^,^20^. The Mtb-DapA structure, determined at 2.28 Å resolution^11^, revealed a homotetramer state, a dimer of tight dimers. The ping-pong reaction mechanism of DapA in *Escherichia coli* is well characterized^21^. Pyruvic acid forms a Schiff base upon condensation with ε-amino group of the active site Lys161 of *E. coli* DapA, and ASA binds to Arg138 located at the entrance of the active site via hydrogen bonding^22^. The aldol condensation between the pyruvate-bound enzyme and ASA is facilitated by a proton relay motif comprised of two tyrosine residues Tyr107 and Tyr133 and a threonine residue Thr44 to yield DHDP^23^. The corresponding amino acid residues in Mtb-DapA are Lys171, Arg148, Tyr117, Tyr143 and Thr54.

Transposon mutagenesis experiments in *M. tuberculosis* showed that the genes of the DAP pathway, namely, aspartokinase (*ask*), aspartate-semialdehyde dehydrogenase (*asd*), dihydrodipicolinate synthase (*dapA*), tetrahydrodipicolinate N-succinyltransferase (*dapD*), N-succinyldiaminopimelate aminotransferase (*dapC*), succinyl-diaminopimelate desuccinylase (*dapE*), diaminopimelate epimerase (*dapF*), and diaminopimelate decarboxylase (*lysA*) are all essential for mycobacterial growth^24^,^25^,^26^ except for *dapB* (dihydrodipicolinate reductase). The *dapB* mutant grows, albeit slowly^27^,^28^. In a recent study, *dapF* was found to be co-expressed with other essential genes including *Rv1611* (tryptophan synthase C), *Rv1308* (ATP synthase A), *Rv2152c* (*murC*, UDP-N-acetylmuramoyl-L-alanine synthetase), and *Rv3028c* (*etpfA*, electron transfer flavoprotein alpha subunit) in *in vitro* growth conditions^29^. Although the *murC* and *dapF* genes are located 628.28 kb apart in the genome of Mtb, a strong positive co-expression of *dapF* with the peptidoglycan pathway gene (*murC*) suggests a co-regulated relationship between the DAP pathway and the peptidoglycan biosynthesis pathway.

Mtb-DapA is a putative drug target for which no potent inhibitor is reported. Couper *et al.*^30^ tested a series of derivatives of piperidine and pyridine-2,6-dicarboxylate against the *Escherichia coli* DapA, including N-oxide of dipicolinic acid and di-imidate of dimethyl pyridine-2,6-dicarboxylate, each with an IC_50_ value of 0.2 mM^30^. Karsten *et al.*^21^ reported a few competitive inhibitors against *E. coli* DapA, which are structural analogues of pyruvate, namely, 3-fluoropyruvate (*K*_*i*_ = 0.22 mM), α-ketobutyrate (*K*_*i*_ = 0.83 mM), α-ketovalerate (*K*_*i*_ = 0.7 mM) and glyoxylate (*K*_*i*_ = 0.016 mM)^21^. Boughton *et al.*^31^ reported 8 conformationally constrained diketopimelic acid analogues as inhibitors and obtained inhibition in the range of 2–49% at 5 mM concentration of each inhibitor^31^. Turner *et al.*^32^ designed inhibitors based on oxygen functionality at the C4 position (–OH or –C=O) which mimics acyclic enzyme-bound intermediate 4-hydroxytetrahydrodipicolinate (HTHDP), but they showed only 50% inhibition with 0.5–50 mM concentrations^32^.

In this work, we tested several pyruvate analogues and investigated the mechanism of inhibition of Mtb-DapA by α–ketopimelic acid. In addition, we also describe the co-crystal structure of Mtb-DapA with α-ketopimelic acid. Our work could serve as a foundation for developing new drug-like inhibitors of Mtb-DapA.

## Results

### Screening of pyruvate analogues

We determined the Michaelis constant, *K*_*m*,_ of the recombinant Mtb-DapA (Mtb-rDapA) for pyruvate and ASA to be 148.8 μM and 388 μM respectively, which are very close to those reported previously^11^. We carried out all assays at fixed concentrations of the substrates, pyruvate and ASA at 500 μM and 400 μM, respectively (Supplementary Figure S3A,B).

**Figure 1:**
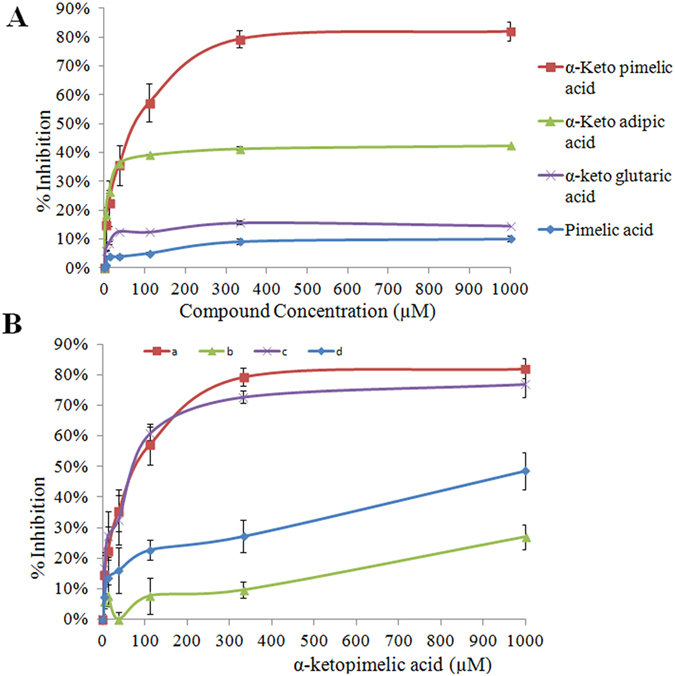
(**A**) IC_50_ analysis of pyruvate analogues: The percentage of inhibition by each inhibitor relative to control (in absence of inhibitor) was determined using the coupled assay. The protein was pre-incubated with each compound for 5 min at various concentrations (0–1000 uM) before the assay. Triplicate measurements were taken at each time point and data were plotted with mean ±1SD. (**B**) Competition analysis to infer the potential binding site of α-KPA: The competition experiments were conducted using the coupled assay after 5 min pre-incubation across various concentrations of α-KPA. Substrates concentrations were held constant at 500 μM of pyruvate and 400 μM of ASA in assay mix. The Mtb-rDapA protein was pre-incubated with (**a**) α-KPA (0-1 mM), (**b**) α-KPA + pyruvate (500 μM), (**c**) with α-KPA + ASA (400 μM), and (**d**) pyruvate (500 μM) before addition of other assay components. Each concentration point was measured at least in triplicate and data were plotted as mean ± 1SD.

The results of kinetic experiments using a coupled assay with various pyruvate analogues are shown in Table 1. The proportion of inhibition measured at different concentrations of the analogues showed that the IC_50_ for α-KPA was 21 μM and the inhibition constant K_i_ was 32.5 μM at 500 μM pyruvate and 400 μM ASA (Fig. 1A). Among the structurally similar compounds (alpha-keto adipicacid, alpha-ketoglutaric acid and pimelic acid) with saturated hydrocarbon chains, only α-KPA could achieve maximal inhibition of 88%. Alpha-ketoadipic acid showed moderate inhibition at 40% and alpha-ketoglutaric acid showed low inhibition at 15% (Fig. 1A). Interestingly, pimelic acid, which is devoid of the α-keto group but retains the same carbon chain length, did not exhibit inhibition even at millimolar concentrations (Fig. 1A). The methyl este,r methyl 3-oxohexanoate, with the keto group shifted by one carbon also did not show any inhibition (Supplementary Figure S4A). These results highlight the critical importance of the α-keto group and the length of the aliphatic carbon chain. We also tested compounds with aromatic groups, namely, benzoic acid, fusaric acid, *p*-amino benzoic acid, *p*-aminosalicylic acid, and phenoxyacetic acid (Supplementary Figure S4B–F). None of these showed inhibition.

Shortening the aliphatic chain of α-KPA by even one carbon had a pronounced effect, with a 50% loss of inhibition. Further chain shortening resulted in further loss of inhibition to 15%. It is interesting to note that the three compounds with any observable inhibitory activity had the highest topological polar surface area 91.7 Å^2^ (Table 1) compared with rest. It is therefore probable that bulkier moieties (aromatic groups) and molecules with lower polar surface area for similar molecular weight may not be as inhibitors of Mtb-rDapA.

### Structural analogues of α-KPA

We carried out a rational design approach for Mtb-DapA inhibitors based on the structure of α-KPA. α-KPA has two COOH groups and a keto group. We carried out a series of substitutions including substitution of the α-keto group with a hydroxyl, substitution of carboxyl groups with amide, and sulfoxides, sulfones, sulfonamides, imidazole and an azole group in the carbon skeleton. We have synthesized a total of 29 molecules. The TPSA varied from 72 Å^2^ to 126 Å^2^ and the molecular weights ranged from 174 Da to 237 Da. Maximum inhibition at 250 μM was in the range of 12–74% (Table 1). The α-hydroxyl pimelic acid was the strongest inhibitor, with a maximum inhibition 74% and IC_50_ of 31 μM (Supplementary Figure S5A,B).

### Competition at the active site

In order to identify the binding site of α-KPA, we determined the maximal inhibition of Mtb-rDapA in various conditions by pre-incubating Mtb-rDapA with (i) α-KPA, (ii) α-KPA and pyruvate, (iii) α-KPA and ASA, and (iv) pyruvate. We observed maximal inhibitions of 82%, 27%, 77%, and 49%, respectively (Fig. 1B). The IC_50_ of Mtb-rDapA pre-incubated with pyruvate was 160 μM, which is nearly 8 fold higher compared to pre-incubation with α-KPA. These results suggest that α-KPA competes with pyruvate for a binding site in Mtb-rDapA.

In order to investigate the stability of the inhibition by α-KPA we carried out competition experiments with varied concentrations of pyruvate ranging from 0.17 to 2 mM and determined the IC_50_ values in each case over different time intervals. ASA was kept constant at 400 μM. The results shown in Table 2 demonstrate that the IC_50_ of α-KPA increases from 21 μM to 61 μM at a high concentration 2 mM of pyruvate affirming that α-KPA is binding to the pyruvate binding site and is competing with it. Further, the gradual increases in IC_50_ with time at all the concentrations of pyruvate tested suggest that the binding of α-KPA to Mtb-rDapA is relatively stable.

### Co-crystallization with α-KPA

**Figure 2:**
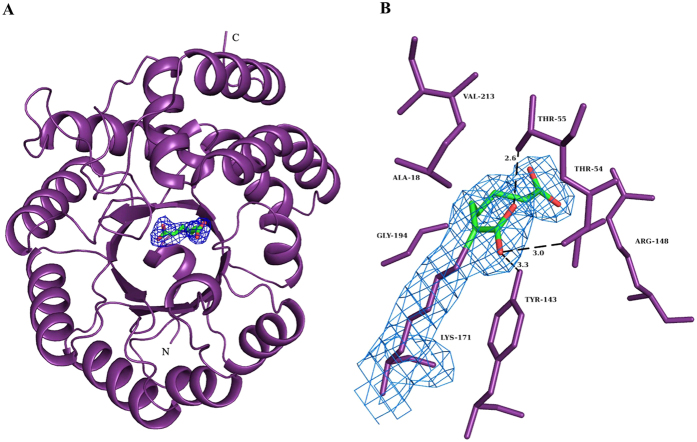
Crystal structure of Mtb-rDapA α-ketopimelic acid complex. (**A**) A monomeric unit of Mtb-rDapA in complex with α-ketopimelic acid. (**B**) An (mFo-DFc) electron density map at 1σ shows the presence of α-ketopimelic acid at the active site of Mtb-rDapA. Residues present at a distance of ca 3.5 Å from the bound inhibitor are displayed.

The structure of Mtb-rDapA in complex with α-KPA was solved at 2.4 Å resolution (Fig. 2A). Except for the first three residues at the N-terminus and one residue at the C-terminus, the entire polypeptide could be modeled unambiguously into electron density (Fig. 2B). The N-terminal region is comprised of an (α/β)_8_ TIM barrel domain and the C-terminal region adopts a three- helical-bundle conformation. It is reported that in Mtb-DapA, Tyr143 and Thr54 of one monomer and Tyr117 of the adjacent monomer constitute the catalytic triad^11^. These residues juxtapose Lys171, a residue that forms Schiff base with pyruvate, and completes the active site boundary^12^,^18^. The bound substrate then undergoes aldol condensation with ASA aided by a proton relay motif comprising Thr54, Tyr117 and Tyr143 to generate DHDP^23^. The disruption of interactions between these amino acids has been proposed as the basis for rational inhibitor design^11^.

The Mtb-rDapA-α-KPA complex structure comprises a monomer of the polypeptide in P4_2_2_1_2 space group (Table 3). Hence, the residue Tyr117 that completes the catalytic triad could not be located closer to the active site. The structure reveals the presence of α-KPA at hydrogen bonding distance from the residues Thr54, Tyr143 and Lys171. Alpha-KPA occupies the position of the substrate pyruvate and thus could prevent the formation of a Schiff base with pyruvate, which is the first step of the ping-pong reaction. In addition, α-KPA also forms hydrogen bonding interactions with Thr55, Arg148 and Gly194. The Mtb-DapA and Mtb-rDapA-α-KPA complexed structures superpose very well with an RMSD of 0.3 Å, indicating an apparent lack of conformational changes in the protein upon ligand binding.

### Thermal shift assay

The binding of pyruvate and α-KPA to Mtb-rDapA was probed by performing a thermal shift assay. Increase in the Tm of a protein in the presence of a ligand is often used as a measure of binding. Mtb-rDapA showed a Tm of 84.8 °C. The Tm increased by 2 °C upon binding pyruvate, and by 1 °C upon binding α-KPA (Supplementary Figure S6).

### Mass spectrometric analysis with MRM

**Figure 3:**
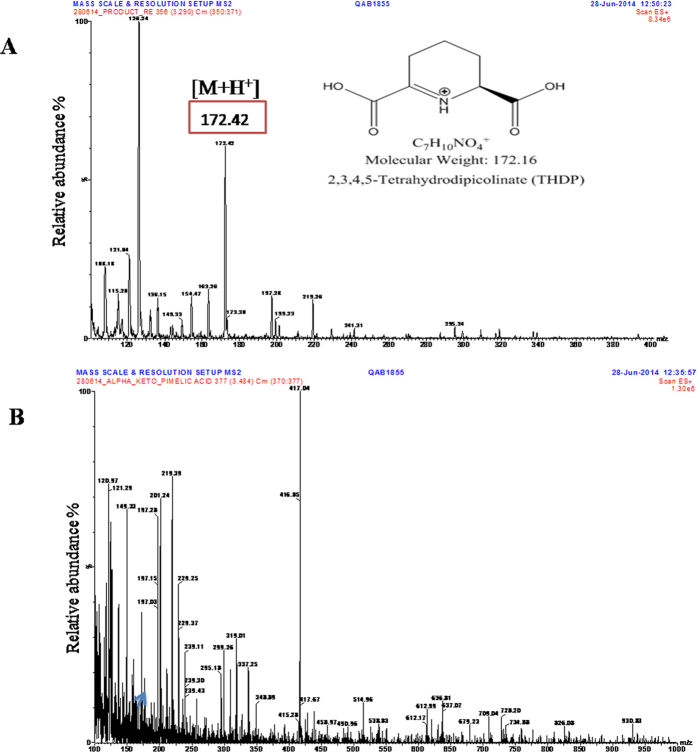
Mass spectral analysis of the reaction mix of Mtb-rDapA and Mtb-rDapB coupled assay mix. (**A**) MS analysis of the Ultra Performance Liquid Chromatography (UPLC)-separated peak (encircled) corresponding to THDP. (**B**) Coupled assay mixture prepared for MS analysis with addition of α-KPA and subjected to UPLC. Small peak (marked arrow) corresponding to THDP mass of 172 was observed.

In order to examine the product formation and inhibition more directly, we carried out LC-MS analyses (Fig. 3). We observed the peak corresponding to THDP ([M + H^+^] = 172.4) when α-KPA was absent and in the presence of α-KPA the relative abundance peak corresponding to the mass of THDP was decreased. These results showed inhibition of the THDP product formation by α-KPA (Fig. 3A,B). In the MS/MS method, THDP was fragmented into two masses 107.4 and 125.3, as evident from a daughter scan corresponding to the parent mass of THDP (Supplementary Figure S7A). The negative control experiment in the absence of ASA did not show any peak corresponding to the THDP mass (Supplementary Figure S7B).

Further, we quantitated the mass of the THDP product using multiple reactions monitoring (MRM) which specifically analyzed the given masses of precursor ion 172.3 and the product ions 125.3 and 107.4 by computing the SRM peak area of the UPLC chromatogram (Supplementary Figure S7C,D). We obtained a large peak area for the THDP product when α-KPA was absent whereas the SRM peak area was reduced by 50% in the presence of α-KPA on account of inhibition of the THDP product formation by α-KPA.

### Mtb-rDapA oligomerization

**Figure 4:**
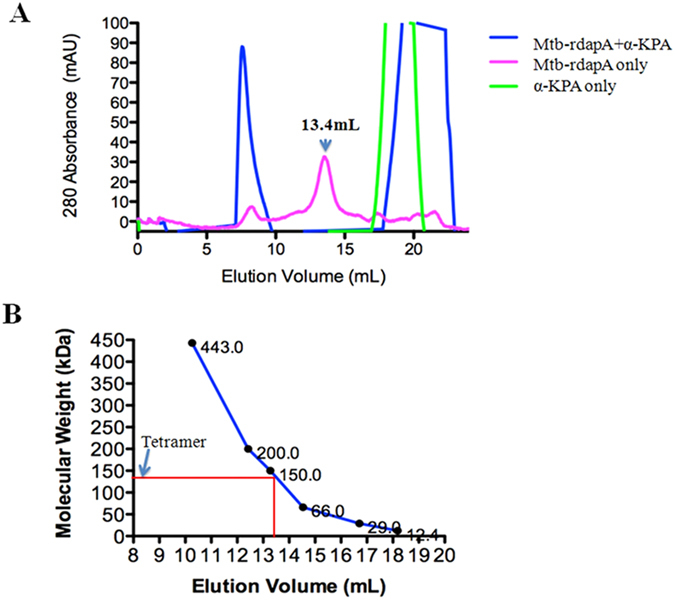
Size exclusion chromatograms of purified Mtb-rDapA. (**A**) Size exclusion chromatogram showed the elution peak of α-KPA in the range 18 to 20 ml fraction (green line). Mtb-rDapA incubated with α-KPA (1:50 ratio) at 37 °C for 5 min resulted in two peaks one at 8 to10 ml elution fraction corresponding to Mtb-rDapA aggregates (blue line) and second one at 19 to 22 ml fraction corresponding to α-KPA (blue line). Mtb-rDapA itself exhibited a peak at 13.4 ml (144 kDa) that corresponds to the tetramer of Mtb-rDapA tetramer (pink line). (**B**) Calibration curve for the size exclusion chromatogram using molecular weight standards proteins, namely, Apoferritin (443 kDa), β-Amylase (200 kDa), Alcohol dehydrogenase (150 kDa), BSA (66 kDa), carbonic anhydrase (29 kDa), cytochrome C (12.4 kDa).

The native Mtb-rDapA formed a tight tetramer in solution with a molecular weight corresponding to 144 kDa. The monomer peak could not be observed (Fig. 4A). Mtb-rDapA was also studied in the presence α-KPA at ratios of 1:5, 1:10 and 1:50 molar excess (Fig. 4A) incubated at 37 °C for 5 min. We did not obtain any peak corresponding to either the tetramer or the octamer of Mtb-rDapA when α-KPA was present in 50-fold molar excess. Only a strong peak corresponding to the α-KPA was observed (Fig. 4). However at ratios of 1:5 and 1:10 of Mtb-rDapA to α-KPA the oligomerization was not affected (Supplementary Figure S8A,B). These results show that in the presence of the inhibitor α-KPA, Mtb-rDapA is unable to form oligomers and this disruption explains the absence of the enzyme activity (Fig. 4A). These observations are also supported by co-crystal structure data, which showed only monomers.

### Docking and Molecular Dynamics Simulation of Mtb-DapA

**Figure 5:**
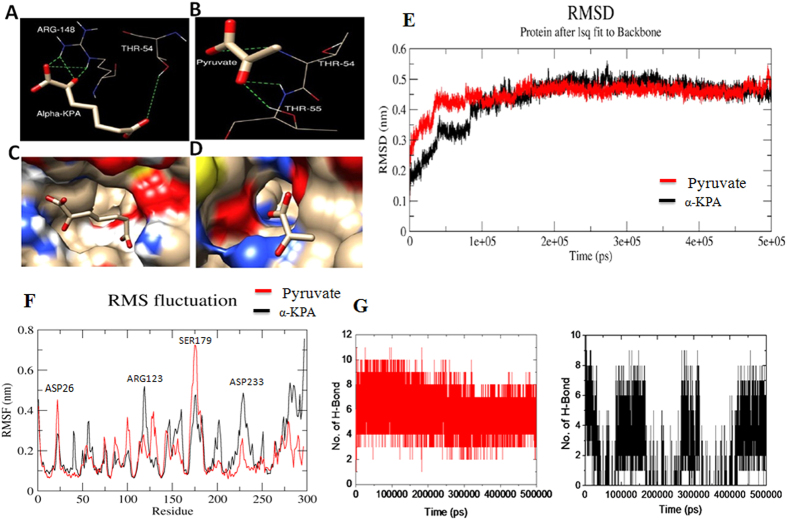
A close view of active site region of Mtb-DapA docked with pyruvate and α-KPA. For docked α-KPA-Mtb-rDapA complex, the starting atom coordinates were taken from the co-crystal structure. (**A**) The H-bond network of α-KPA derived from Molecular Docking with Mtb-rDapA as the template is shown with active site residues. (**B**) Docked complex with substrate pyruvate. (**C**) The molecular surface of Mtb-rDapA showing the orientation of α-KPA (**D**) orientation of substrate pyruvate. (**E**) Cα backbone RMSD plots of MD simulation of ligand-Mtb-DapA. The α-KPA Mtb-rDapA complex is shown in black contour and pyruvate Mtb-rDapA complex is shown in red contour. X-axis shows the simulation time in picoseconds and y-axis shows the backbone RMSD in nanometers. (**F**) Root mean square fluctuation (RMSF) of amino acid residues of inhibitor Mtb-rDapA complex: α-KPA Mtb-rDapA complex RMSF plot is shown as black trace and pyruvate-Mtb-DapA complex RMSF plot is shown as red trace. (**G**) Plots representing the formation of hydrogen bonds between the ligands and Mtb-rDapA during the entire simulation: α-KPA-Mtb-rDapA complex is shown in black lines, pyruvate-Mtb-DapA complex is shown in red lines.

The crystal structure of native Mtb-DapA (PDB ID: 1XXX)^11^ was used as a template and molecular docking with ligands were carried out with Autodock VINA^33^. The binding free energy of the substrate pyruvate, α-KPA and other ligands upon binding to the Mtb-DapA active site were estimated. The docked poses and lowest binding free energy for each of the ligands were evaluated. Among the docked ligands, binding of α-KPA (−5.1 kcal/mol) and pyruvate (−2.8 kcal/mol) were observed to be consistent within the active site of Mtb-DapA. The hydrogen bond network at the active site residues of Mtb-DapA docked with α-KPA and pyruvate are shown in Fig. 5A–D.

In order to examine the overall stability of the binding mode we carried out molecular dynamics (MD) simulations of the docked complexes (1) Mtb-DapA + α-KPA and (2) Mtb-DapA + Pyruvate. Initial simulation experiments showed that the ligand α-KPA drifted away from the active site pocket within 1 ns and never returned to the active site over the 100 ns simulation, in contrast to pyruvate, which remained bound throughout. Therefore, in the case of α-KPA we repeated simulations using the co-crystal complex.

We performed MD simulations of α-KPA and pyruvate-docked to Mtb-rDapA using GROMOS 53 force field up to 500 ns. The stability and per-residue fluctuations were monitored by calculating root mean square deviation (RMSD) of the MD trajectory. As depicted in Fig. 5E, the RMSD of both the docked complexes increased initially due to the relaxation of the structure from the starting conformation and both models were stabilized during the entire simulation time. The RMSD of all the backbone atoms of α-KPA-docked Mtb-rDapA (black contour) reached a plateau after 40 ns whereas the RMSD of all the backbone atoms of pyruvate-docked Mtb-DapA (red contour) reached a plateau after 90 ns (Fig. 5E).

To identify the regions of higher flexibility, the per residue root mean square fluctuation (RMSF) was calculated during the last 400 ns (Fig. 5F). The RMSF of the α-KPA-bound Mtb-rDapA is shown in the black contour, whereas that of the pyruvate bound model is shown in the red contour.

As is evident, the RMSF of the N-terminal regions, encompassing amino acid residues 20–30, 80–102, and 172–185, exhibited high fluctuation, ranging from 0.38 nm to 0.7 nm in the pyruvate-Mtb-DapA complex compared to the α-KPA-bound complex. On the other hand, Arg123 in the N-terminal half and the entire α-helix portion of the C-terminal region, encompassing amino acid residues 200–300, were observed to be highly fluctuating (up to 0.7 nm) in the α-KPA-bound complex (black contour) compared to the pyruvate-docked complex (red contour). Interestingly, perhaps due to hydrogen bond formation of the ligand (α-KPA or pyruvate) with the active site residues Thr54, Thr55, Tyr143, Arg148 and Lys171, these residues were not fluctuating. It is clearly evident from the differences in the RMSF of the amino acid residues of two cases that, even though α-KPA and pyruvate bind in the active site region of Mtb-DapA, the molecular dynamics results show significant differences in the interactive modes between the two ligands.

The hydrogen bonding patterns between ligands and Mtb-DapA or Mtb-rDapA during simulations suggest (Fig. 5G) that pyruvate maintains stable hydrogen bonding with the active site residues throughout the simulation, in contrast to α-KPA. Although the two simulations are not strictly comparable, these results offer an approximate view of the differences between the binding properties of the two ligands. Likewise, in the case of structural analogues of α-KPA, only the α-hydroxyl pimelic acid showed consistent hydrogen bond during simulation (Supplementary Figure S9).

## Discussion

Previous studies of the co-crystal structure of *Escherichia coli* DapA complex with the inhibitor alpha-ketopimelic acid (α-KPA) had shown that α-KPA interacts with the pyruvate binding site^8^. We obtained a similar result, thereby validating α-KPA as an inhibitor candidate for Mtb-rDapA.

In order to test the role of the different moieties of α-KPA, considering α-KPA as the base inhibitor, we designed several analogues, either varying the chain length or eliminating the α-keto group. We observed that the α-keto group is essential for inhibition. Shortening the chain length even by one carbon atom decreases the maximal inhibition drastically up to 50%, even with retention of the α-keto group. Compounds containing aromatic groups had no observable inhibition of Mtb-rDapA (Table 1). Similarly, the substitutions of an amide or ester at the carboxylic acid end of α-KPA could not improve the inhibition compared with α-KPA. However, replacement of the keto group with a hydroxyl moiety achieved inhibition comparable with α-KPA (Table 1). It is noteworthy that for the similar molecular weight range, the topological polar surface area 91.7 Å^2^ plays a cardinal role in inhibition.

The IC_50_ of α-KPA did not remain constant at varying pyruvate concentrations (0.17–1 mM) although in the initial 30 minutes it is stable. These experiments showed that with time the IC_50_ increased up to 2 fold showing that the binding of the α-KPA with Mtb-rDapA, although stable, can be overcome by competition over time or by increasing concentrations of pyruvate. In the case of pyruvate, the Schiff base condensation with pyruvate could pull the equilibrium towards the Mtb-rDapA pyruvate complex. As in the case of *E. coli*, in Mtb-rDapA, α-KPA binds in the same active site as pyruvate. These data are supported by co-crystal structure data as well. Direct examination of inhibition of THDP product formation by LC-MS analysis, showed up to 50% reduction in area under the curve (AUC) inhibition at 1 mM KPA (saturating concentration) by multiple reaction monitoring analyses (Fig. 3D,E). Most DapA enzymes from bacteria have been shown to exist as homotetramers and such a quaternary structure appears to be required for the enzyme activity^11^. Our results show that α-KPA disrupts the oligomerization of Mtb-rDapA thereby causing loss of enzyme activity. Thus, the paradigm of disruption of tetramer structure of DapA underlying inhibition of enzyme activity is evident from these results.

Molecular dynamics simulations over 500 ns showed that pyruvate was consistently bound to the active site showing stable binding through hydrogen bonds throughout the entire simulation whereas α-KPA exhibited a less stable binding mode (Supplementary Figures S10 and S11). These data indicate that α-KPA binding to the enzyme requires a specific stereo-chemical orientation in order to achieve maximum and stable binding. Therefore ligands with specific stereo chemically locked conformation to bind to active site could be preferred inhibitors.

Published reports on the emergence of Mtb resistant to first and second line drugs including isoniazid, rifampicin, pyrazinamide, ethambutol, streptomycin, cycloserine, and moxifloxacin highlight the complexity of current TB drug regimens. Some of these drugs increased the risk of hepatotoxicity, fever, nausea and skin rashes. We hope that our report describing a new set of inhibitors could aid the discovery pipeline of new TB drugs^34^.

## Conclusions

1. α-ketodicarboxylic acid of chain length (7 carbons) is an inhibitor of Mtb-rDapA.
2. The substitution of amide or ester at the carboxylic acid end inhibited the enzyme in the range of 45–65%.
3. Contraction and extension of the carbon chain length with inclusion of other substituents did not preserve or improve inhibition.
4. The α-keto group is essential to achieve maximal inhibition. This group can be reduced to hydroxyl.
5. α-KPA binds to the substrate (pyruvate) binding site.
6. This competitive binding is supported by co-crystal structure data.
7. Inhibition is achieved by disruption of the tetrameric structure of Mtb-rDapA.

## Methods

### Cloning, expression and purification of DapA

#### Polymerase chain reaction (PCR) amplification and cloning

The Mtb-*dapA* and *dapB* open reading frames were cloned in the expression vector pET28a (Novagen, USA) for expression as N-terminal 6x His-tagged proteins. The genomic DNA of *M. tuberculosis* H37Rv was used as the template in the PCR amplification using the following primers: Forward primer for *dapA* 5′-AACCTTGGGATCCGTGACCACC–3′ and Reverse primer was 5′-GGGAAGGTCTCGAGCCACTTCTGGG-3′. forward primer for *dapB* was 5′-GTCTAGGGGATCCGCCATGCGGGTA-3′ and the reverse primer 5′-TGAACGCGATTAT CAACTCGAGATACAGG-3′. In both cases the restriction sites *BamHI* and *XhoI* were added in the forward and in the reverse primer respectively. The PCR conditions were initial denaturation step of 5 min at 95 °C followed by 30 cycles of 30 sec at 95 °C, 45 sec at 53 °C (for *dapA*) or 59 °C (for *dapB*), 90 sec at 72 °C and a final extension step of 10 min at 72 °C, using Taq DNA polymerase (Bangalore Genei, India). The PCR product was gel eluted and purified using DNA isolation kit (Qiagen, Germany). PCR products were double digested with the restriction enzymes *BamHI* and *XhoI* and then cloned in frame in pET28a predigested with the same restriction endonucleases (New England Biolabs (NEB), USA) ligated using T4 DNA ligase (NEB, USA). Recombinant plasmids were transformed in to *E. coli* DH5α, selected by colony PCR screening and confirmed by sequencing.

#### Over-expression and purification of recombinant protein

To over-express the fusion constructs, plasmids were transformed into *E. coli* BL21(DE3) (Invitrogen, USA) and transformants were grown at 37 °C in Luria Bertani Broth (Himedia Laboratories, India) containing 50 μg/ml Kanamycin. At an optical density of 600 nm (OD_600_) of 0.6, the culture was cooled to 16 °C in ice water for 1 hour, induced by adding Isopropyl-D-thio-β-galactopyranoside (IPTG; Sigma, USA) to a final concentration of 0.25 mM and incubation was continued with shaking (220 rpm) for 16 hours at 16 °C. The cells were harvested by centrifugation at 6800 × g for 10 min at 4 °C. The pellet was resuspended in 10 ml of lysis [20 mM Tris-HCL, pH 7.9, 500 mM NaCl, 10 mM imidazole, 5% (v/v) glycerol, 2 mM β-mercaptoethanol, 1 mM phenyl methyl sulfonyl fluoride (PMSF; Sigma-Aldrich, USA)].

Lysozyme (Hen egg white, Sigma, USA) was added in the suspension to final concentration of 1 mg/ml and incubated for 1 hour on ice. Cells were lysed by sonication (Sonic Vibra-Cell) with 30 cycles of 30 s pulse and 20 s rest on ice followed by centrifugation at 29000 × g for 30 min at 4 °C. Expressed proteins (Mtb-rDapA and Mtb-rDapB) were found to be soluble by analyzing supernatant and pellet fractions on 12% SDS-PAGE (Laemmli, U.K. 1970). Purification of 6x His-tagged proteins, Mtb-rDapA and Mtb-rDapB, were carried out by affinity chromatography using Nickel-Nitrilotriacetate (Ni-NTA) resin column (Qiagen, Germany). The soluble fraction was incubated with 1 ml of Ni-NTA resin pre-equilibrated with equilibration buffer (20 mM Tris-HCl, pH 7.9, 500 mM NaCl) overnight at 4 °C. The supernatant was loaded on to the column and washed with 10 column volumes of wash buffer-1 (20 mM Tris-HCl, pH 7.9, 500 mM NaCl, 5 mM imidazole, 5% (v/v) glycerol, 2 mM β-mercaptoethanol), 25 column volumes of wash buffer-2 (20 mM Tris-HCl, pH 7.9, 500 mM NaCl, 20 mM imidazole, 5% (v/v) glycerol, 2 mM β-mercaptoethanol), five column volumes of wash buffer-3 (20 mM Tris-HCl, pH 7.9, 500 mM NaCl, 50 mM imidazole, 5% (v/v) glycerol, 2 mM β-mercaptoethanol), five column volumes of wash buffer-4 (20 mM Tris-HCl, pH 7.9, 500 mM NaCl, 100 mM imidazole, 5% (v/v) glycerol, 2 mM β-mercaptoethanol).

Elution of bound proteins were carried out with elution buffer (20 mM Tris-HCl, pH 7.9, 500 mM NaCl, 250 mM imidazole, 5% (v/v) glycerol, 2 mM β-mercaptoethanol). The fractions containing significant amount of purified Mtb-rDapA or Mtb-rDapB as observed by SDS-PAGE (12%) analysis were pooled and dialyzed against 20 mM Tris-HCl, pH 7.9, 500 mM NaCl, 5% (v/v) glycerol using dialysis membrane (3.5K MWCO, ThermoFisher SCIENTIFIC, USA) at 4 °C overnight and concentrated using Amicon ultra concentrator-15 (3 kDa NMWL) (Millipore, USA), and stored at 4 °C. The concentrations of recombinant proteins were estimated by Bradford method (Bio-Rad Laboratories, USA) using known concentration of BSA (Sigma-Aldrich, USA) as standard.

### Western Blotting and MALDI-TOF analysis

Purified Mtb-rDapA and Mtb-rDapB proteins were electrophoresed on SDS-PAGE (12%) at 100 V, 90 mA for 3–4 hours in Mini-PROTEIN Tetra Cell (Bio-Rad, USA) at room temperature. For size determination, Pre-stained molecular weight standards (10–170 kDa) (Fermentas, Canada) was used. Transfer of proteins from SDS-PAGE to nitrocellulose membrane (Bio-Rad, USA) was carried out using a TransBlot apparatus (Bio-Rad, USA) in the transfer buffer (24 mM Tris-HCl pH 8.0, 192 mM glycine and 20% methanol) for 2 hours at 90 V at 4 °C^35^. Ponceau S (Sigma, USA) dye at final concentration (0.1%) was used to confirm transfer. After washing the Ponceau S stain using PBS buffer (137 mM NaCl, 2.7 mM KCl, 4.3 mM Na_2_HPO_4_, and 1.47 mM KH_2_PO_4_) the blot was incubated in blocking buffer containing 5% Skimmed milk powder (Sigma, USA) for 2 hours at room temperature. Blots were washed twice with PBS-T (PBS + 0.2% Tween-20) and PBS, and were incubated with horseradish peroxidase (HRP) conjugated anti-Histidine antibodies (Qiagen, Germany) at 1:5000 dilution for 2 hours at room temperature. Finally, the blot was washed twice with PBS-T and PBS. The blot was developed using 0.05% of 3, 3′-Diaminobenzidine (DAB) (Sigma, USA) (Supplementary Figure S12).

In order to further confirm identity of purified recombinant proteins, peptide mass fingerprinting was carried out using trypsin and the tryptic digested peptides. The ‘mass spectra’ were searched using MASCOT search engine against MSDB (mass spectrometry protein sequence Database)^36^.

### Coupled enzymatic activity assay

The purified N-terminal 6x His-tagged Mtb-rDapA and Mtb-rDapB proteins were used to prepare the coupled enzyme assays (Supplementary Figure S12). The coupled enzyme assay was used to investigate the kinetics of Mtb-rDapA. The formation of dihydrodipicolinate by Mtb-rDapA was measured by reduction to tetrahydrodipicolinate by Mtb-rDapB using NADPH as electron donor. The consumption of NADPH can be measured spectrophotometrically at 334 nm. The following components were prepared fresh in each experiment at final concentration: pyruvate (500 μM), Aspartate semi aldehyde [(*S*)-ASA] (400 μM) and NADPH (160 μM). (*S*)-ASA in isolated form is unstable except in aqueous strong acid. Therefore we synthesized the precursor of (*S*)-ASA known as compound 7 (tert-butyl derivative)^37^ commercially, which was of high quality (>95%) as judged by ^1^H-NMR. Just before the assay, compound 7 was deprotected using trifluoroacetic acid and solvent 1,2-dichloroethane (1:1 v/v) and stored on ice^37^. The standard assay for Mtb-rDapA activity was performed in 50 mM sodium phosphate buffer pH 7.4 containing NADPH (160 μM), Mtb-rDapA (0.2 μg/ml, 36 nM) and Mtb-rDapB (3 μg/ml, 0.58 μM). The concentration of pyruvate was varied from 170 μM to 2000 μM and readings were taken in triplicate and the experiments were repeated until consistent kinetics was obtained. Similarly the concentration of substrate ASA was varied from 35 μM to 4000 μM until consistent kinetics was obtained.

Assay mix (92 μl) was added to each well in a 96 well plate (Tarson, India) and incubated at 37 °C for 5 min. The reaction was started by the addition of the (*S*)-ASA (8 μl). The absorbance at 340 nm was measured at 37 °C (Infinite200PRO spectrophotometer, TECAN, Europe) over a time period of 600s (10 min). Water was used as blank and the negative control contained assay mixture without (*S*)-ASA. Decrease in absorption commensurate with decreasing concentration of NADPH corresponded to the consumption of substrate, or equivalently, formation of product. The Mtb-rDapB was 16 times in molar excess to ensure complete consumption of dihydrodipicolinate formed. All kinetics measurements were performed in triplicate in 96 well plates (Tarson, India), and all experiments were repeated several times to ensure that reproducible results were obtained. Percentage of inhibition was calculated from the formula, % of inhibition = ([(A_control_ − A_test_)/A_control_]) × 100, where A_control_ is the absorbance of the control sample and A_test_ is the absorbance of the test sample. The IC_50_ was calculated using nls package in R to the Michaelis–Menten type equation by formula, V2 ~ a*V1/(b + V1), where V2 = % Inhibition, V1 = Inhibitor Concentration, a = Maximum Inhibition, b = IC_50_ (Inhibitor Concentration at half Inhibition). In the pre-incubation experiments, initially Mtb-rDapA was pre-incubated at 37 °C for 5 min with varying concentrations of the inhibitors. At predetermined time intervals, aliquots were transferred to the standard assay mixture and assayed for enzyme activity.

Thus, the activity was measured by following the decrease in absorbance at 340 nm corresponding to the oxidation of NADPH to NADP^+^. This is easily quantifiable because, while NADPH has a strong absorbance at 340 nm (the extinction coefficient, ϵ_340′_ is 6.3 × 10^3 ^M^−1^cm^−1^) NADP^+^ exhibits negligible absorbance at this wavelength. This assay is able to measure DapA (Mtb-rDapA) kinetics if DapB (Mtb-rDapB) is present in excess, because under these conditions DapA becomes rate limiting.

### Selection of compounds and their synthesis for inhibition studies

In this work we tested structural analogues of pyruvate, namely, α-KPA, α-ketoglutaric acid, α- ketoadipic acid Pimelic acid, Benzoic acid, Fusaric acid, p-amino Benzoic acid, Methyl butyrylacetate, p-aminosalicylic acid and Phenoxyacetic acid. Various concentrations (0.125 mM, 0.25 mM, 0.5 mM and 1 mM) of these water soluble compounds were used to test the extent of inhibition.

The α-KPA analogues were prepared mainly with substitutions of Amide (C=ONH_2_), Amine (R-NH_2_), Ester (R-COOR), Sulfonamides (S-CONH_2_), Sulfoxide (R-S=OR’), Sulfone group (R-SOOR), hydroxyl group (-OH) and aromatic rings, namely, phenyl, benzene, imidazole. The solid state compounds having molecular weight in the range of 174–237 Da were 90–97% pure as measured by LC-MS data.

In this study the inhibition of Mtb-DapA were tested with 29 structural analogues of α-KPA as listed in the Table 1. 23 were soluble in 100% methanol and rests were soluble in water at 1 mg/ml. We tested inhibition characteristics of the compounds in the concentration ranges of 10 μM, 50 μM, 100 μM, 200 μM and 250 μM.

### Crystallization, data collection and structure solution

Mtb*-*rDapA (6 mg/ml) was incubated with α-ketopimelic acid at two different Mtb-rDapA: α-ketopimelic acid molar ratios (1:10; 1:50) for 2–3 hours at 4 °C. Post incubation, the sample was centrifuged at 6800 × g for 20 minutes. Initial crystallization experiments with the incubated mixture were performed using crystallization screens (Hampton Research, Inc.). Crystallization trials were performed using both the micro-batch method and hanging drop vapour diffusion methods of crystallization. Plate like, poorly diffracting crystals were obtained in the condition: 1.4 M Sodium Citrate Dihydrate, 0.1 M HEPES, pH 7.5 within three weeks of setting up the crystallization experiment. Optimization by varying the concentrations of the components in crystallization condition resulted in single large crystals in the condition containing 1.34 M tri-sodium citrate, 0.1 M Tris-HCl, pH 7.5 at 1:10 of Mtb-rDapA to α-ketopimelic acid ratio (Supplementary Figure S13). Data was collected on one such crystal on a MAR345 image plate mounted on a Rigaku MicroMax007HF rotating-anode X-ray generator. The data were integrated using iMOSFLM^38^ and scaled using SCALA^39^. The phase information was obtained by Molecular Replacement method using PHASER^40^ (*M. tuberculosis* DapA, PDB: 1XXX was used as a search model)^11^. The model was built using COOT^41^ and refined using Refmac5 from the CCP4 suite of programs^42^. The ligand was incorporated into the model at the final stages of refinement. The library files for α-ketopimelic acid were obtained from the PRODRG server (davapc1.bioch.dundee.ac.uk/prodrg). The data collection and refinement statistics are presented in Table 3. Furthermore, we have deposited and validated the PDB in RCSB protein data bank and the given PDB ID is 5J5D.

### Thermal shift assay for inhibitor screening

The fluorescence based thermal shift assay for screening of identified ligands, which is based on the principle that ligand binding alters thermal stability of protein. This method allows us to monitor the protein denaturation upon heating using fluorescence based dye (Sypro orange, Sigma, USA), which binds to hydrophobic part of proteins during protein denaturation and emits fluorescence at 580 nm^43^. We can monitor the changes in fluorescence and generate a thermal melting curve using real time PCR. The mid-point of the melting curve designated as the melting temperature Tm corresponding to 50% of protein being denatured.

The thermal shift assay was carried out in the LightCycler^R^ LC480 qPCR machine using 384 well PCR plates (Roche, USA). An assay mixture of 20 μl contained 20 μM Mtb-rDapA, 15 X Sypro Orange (Sigma), 50 mM sodium phosphate buffer pH 7.4 and ligand in the range 0.2 mM to 1 mM. The ligands and Mtb-rDapA were diluted in sodium phosphate buffer. The PCR plates were sealed with optical seal, mixed well and centrifuged after the assay mixture was added. The thermal scanning was started from 20 °C to 95 °C at a heating rate of 0.5 °C/min. The fluorescence intensity was measured by excitation at 465 nm and emission at 580 nm. Curve fitting, melting temperature calculation and report generation on the raw Fluorescence based thermal shift assay (FTS) data were performed using pre-installed analysis software provided with the instrument (Roche, USA)^44^.

### LC/MS analysis of 2,3,4,5-tetrahydrodipicolinic acid (THDP), product of the coupled assay

The activity of Mtb-rDapA and Mtb-rDapB in catalyzing formation of THDP was confirmed by LC/MS analysis. A reaction mixture contained 1 μg of Mtb-rDapA, 30 μg of Mtb-rDapB, 10 mM pyruvate, 1 mM ASA, 0.16 mM NADPH and 50 mM sodium phosphate buffer pH 7.4 run at 37 °C for 5 min using UV-Vis spectrophotometer for THDP formation. Mtb-rDapA and Mtb-rDapB were then inactivated at 55 °C for 5 min to preserve integrity of reaction products and reaction mixture passed through 10 kDa cutoff filter unit (Millipore, USA) to filter the proteins and centrifuged at 6800 × g. The flow-through containing reaction products was used in LC/MS analysis. In order to observe the effect of inhibitor on THDP formation we added α-KPA in the assay mixture. The sample was subjected to analysis on a LC/ESI-MS (Quattro Micro API, Waters) coupled to a UPLC inlet. The flow through was loaded onto an Acquity UPLC BEH shield RP18, 1.7 mm, 2.1 100 mm column comprising a mobile phase of buffer A (water, 0.1% formic acid) and buffer B (acetonitrile, 0.1% formic acid) at a flow rate of 0.2 ml min^−1^. The same protocol was followed without ASA as a negative control and subjected to LC/MS analysis. The mobile phase gradient program started at 100% of buffer A then decreased to 25% at 6.0 min and held for 7^th^ min, reversed the gradient for 8 min to 100% of buffer A. The mobile phase returned to the initial composition at 8.0 min and equilibrated before the next injection. Electrospray ionisation was performed in the positive ion mode with the following parameters: capillary voltage 3.5 KV, cone voltage 35 V, desolvation temperature 350 °C, nebuliser gas (N_2_) 750 L/hr. For fragmentation of THDP the collision gas (Ar) was used with collision energy 25 eV. Quantitative analysis of THDP formation was carried out by assessment of peak area in multiple reactions monitoring (MRM) mode with precursor ion 172.3 of THDP and corresponding daughter ions 125.6 and 107.5 by using above mentioned LC-MS/MS parameters.

### Size exclusion Chromatography

Prior to gel filtration, the pre-packed Superdex 200 10/300 column (GE Healthcare) was pre-equilibrated with 50 mM Tris HCl pH 7.5, 150 mM NaCl and 10% glycerol. 25 μM Mtb-rDapA in 25 mM Tris/150 mM NaCl buffer was loaded in 500 μl volume. Multiple fractions of 1 ml volume were collected and each fraction runs on 12% SDS-PAGE gel electrophoresis. The ligand α-KPA in 1:5, 1:10 and 1:50 molar ratio of Mtb-rDapA was incubated with Mtb-rDapA and subjected to run as above. The chromatograms were collected by measuring absorption at 280 nm as a function of buffer volume at a flow rate of 0.3 ml/min. The column was calibrated by passing Apoferritin eluted at 10.5 ml, β-Amylase eluted at 12.5 ml, Alcohol dehydrogenase eluted at 13 ml, BSA eluted at 14.5 ml, carbonic anhydrase eluted at 16.9 ml, and cytochrome C eluted at 19 ml followed by a standard curve generation.

### Docking studies

The initial atom coordinates of Mtb-DapA crystal structure were obtained from the RCSB protein data bank (PDB id: 1XXX)^11^ and used to generate the starting coordinates for the docking study. Molecular docking of DapA and its putative inhibitors were carried out using Autodock Vina (version 4.0)^33^, which has significant improvements and average accuracy of the binding mode prediction compared to other docking algorithms^33^ and also allows flexibility in the ligand.

The docking parameters include ligand and receptor preparation involving addition of hydrogen atoms, merging non-polar hydrogen atoms, rotatable torsion bond followed by grid construction with volume of 22 Å × 24 Å × 26 Å spacing of 0.375 Å centered on the receptor active site amino acid residue Cα of LYS 171, x = 9.785, y = 42.020, z = 69.219. All the compounds were screened on the basis of binding energy and hydrogen bonding with active site residues of Mtb-rDapA. Among the active site residues, Thr54, Thr55 and Tyr143 showed interaction with these compounds through hydrogen bonding.

### Molecular Dynamics Simulation

The best fitting conformation of Mtb-DapA or Mtb-rDapA and its ligand (pyruvate or α-kpa) complex obtained from the docking study was further examined using molecular dynamics (MD) simulation to examine the stability of Mtb-DapA and ligand interaction. MD simulation was carried out using GROMOS53a6.ff all atom force field in GROMACS version 4.5.4^45^,^46^ with explicit solvent and under periodic boundary conditions. GROMOS topology file for ligand was obtained from PRODRG (version 2.5)^47^. The starting coordinates of DapA-ligand complex was neutralized with 7 sodium ions and wrapped up in a periodic box of TIP3P water model that was extended to 10 Å from the solvent. The Particle Mesh Ewald method was used to treat the long-range electrostatics^48^ during the MD simulation. The bond lengths within the protein were constrained using LINCS algorithm^49^. Energy minimization was performed using steepest descent method (50,000 steps, 100 ps) in order to relax the initial solvent and ion configuration and to eliminate any residual strain. To maintain the temperature of the system at a constant value of 300 K, a Berendsen thermostat^50^ was applied. The ligand position and protein were restrained and the solvent and ions around were equilibrated in two phase. The first phase was temperature coupling step conducted under the NVT (constant Number of particles, Volume, and Temperature) ensemble for 200 ps to stabilize the temperature of the system at a constant temperature of 300 K. Similarly, the second phase was pressure coupling step to equilibrate the pressure under the NPT (constant Number of particles, Pressure and Temperature) ensemble for 200 ps at a constant pressure of 1 atm. The conformational sampling (MD simulation data collection) was started after the equilibration step at a constant pressure (1 atm) and a temperature (300 K). The time steps of 2 ps were used to collect the MD simulation trajectory.

### Molecular Dynamics trajectory analysis

Root mean square deviation (RMSD) and root mean square fluctuation (RMSF) were calculated by using GROMACS MD simulation package (g_rms and g_rmsf) tools. The RMSD is calculated for every snapshot collected as function of simulation time. In this study RMSD was computed for the backbone atoms of the protein and the ligand, taking the starting coordinates as the reference structure. The RMSF is a measure of the flexibility calculated as the square root of the variance of the fluctuation around the average position for backbone atom of each residue. Details of H-bond interactions between the ligand and the protein were examined using g_hbond module in GROMACS.

## Additional Information

**How to cite this article**: Shrivastava, P. *et al.* Inhibition of *Mycobacterium tuberculosis* dihydrodipicolinate synthase by alpha-ketopimelic acid and its other structural analogues. *Sci. Rep.* **6**, 30827; doi: 10.1038/srep30827 (2016).

## Supplementary Material

Supplementary Information

## Figures and Tables

**Table 1:**
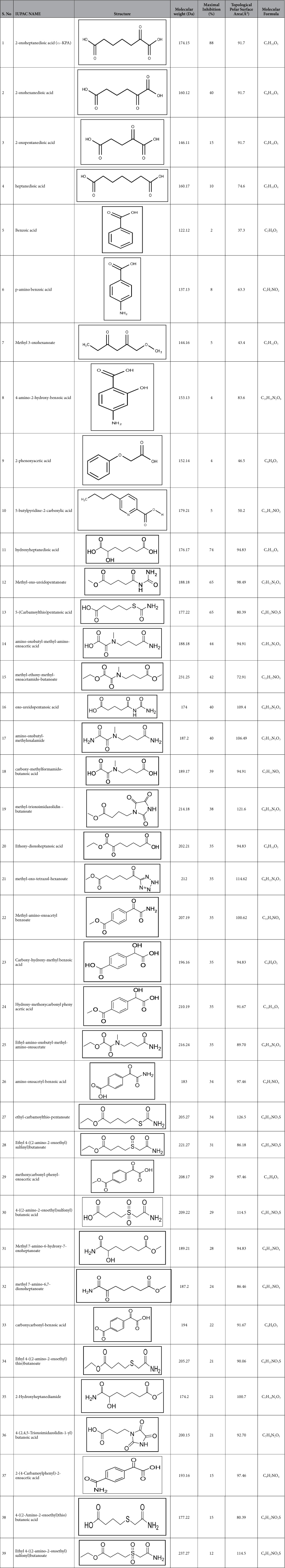
Pyruvate and α-KPA analogues tested for inhibition of Mtb-rDapA.

Maximal inhibition is the highest inhibition achieved relative to that in absence of inhibitor by varying the concentration of the inhibitor at a given concentration of other substrates pyruvate 500 μM and ASA 400 μM.

**Table 2:** IC_50_ of α-KPA.

Time (min) from start	0.17 mM	0.3 mM	1 mM	2 mM
10	21	21	21	61
20	21	21	21	61
30	21	21	21	71
40	31	31	31	81
50	41	41	41	91
60	51	51	51	101

Inhibition of Mtb-rDapA in the presence of 400 μM of ASA and variable concentrations of pyruvate.

**Table 3:** Co-Crystal Data collection, processing and refinement statistics.

Data collection statistics	
Wavelength (Å)	1.5418
Resolution (Å)	2.40 (2.53–2.40)^a^
Space group	P4_2_2_1_2
Cell dimensions (Å)	a = b = 84.78, c = 83.92
R_merge_ (%)^b^	10.6 (42.0)
Total number of observations	83110 (9752)
Total number of unique observations	12296 (1718)
Mean I/σI	13.7 (3.9)
Completeness (%)	98.8 (97.3)
Multiplicity	6.8 (5.7)
Refinement statistics	
R_work_ (%)^c^	19.78
R_free_ (%)^d^	25.39
Number of residues	299
Number of water molecules	97
Ligands	1
RMSD bond length (Å)	0.002
RMSD bond angle (^o^)	0.57
Ramachandran statistics	
Preferred regions (%)	97.3
Allowed regions (%)	2.7
Outliers (%)	0

^a^Values for outer shells are given in parenthesis.

^b^R_merge_ = Σ_j_|<I> − I_j_|/Σ<I> where I_j_ is the intensity of the j^th^ reflection and <I> is the average intensity.

^c^R_work_ = Σ_hkl_|F_o_ − F_c_|/Σ_hkl_|F_o_|.

^d^R_free_ was calculated as for R_work_ but on 5% of the data excluded from the refinement calculation.

## References

[b1] WHO. Global Tuberculosis Report 2014. World Health Organization (2014).

[b2] Smith T., Wolff K. A. & Nguyen L. Molecular biology of drug resistance in Mycobacterium tuberculosis. Curr. Top. Microbiol. Immunol. 374, 53–80 (2013). 23179675 10.1007/82_2012_279 PMC3982203

[b3] Hoffmann C., Leis A., Niederweis M., Plitzko J. M. & Engelhardt H. Disclosure of the mycobacterial outer membrane: cryo-electron tomography and vitreous sections reveal the lipid bilayer structure. Proc. Natl. Acad. Sci. USA. 105, 3963–3967 (2008). 18316738 10.1073/pnas.0709530105 PMC2268800

[b4] Evans G. *et al.* A tetrameric structure is not essential for activity in dihydrodipicolinate synthase (DHDPS) from Mycobacterium tuberculosis. Arch. Biochem. Biophys. 512, 154–159 (2011). 21672512 10.1016/j.abb.2011.05.014

[b5] Kefala G. & Weiss M. S. Cloning, expression, purification, crystallization and preliminary X-ray diffraction analysis of DapA (Rv2753c) from Mycobacterium tuberculosis. Acta Crystallogr Sect F Struct Biol Cryst Commun 62, 1116–1119 (2006). 10.1107/S1744309106039844 PMC2225217 17077492

[b6] Gokulan K., Rupp B., Pavelka M. S., Jacobs W. R. & Sacchettini J. C. Crystal structure of Mycobacterium tuberculosis diaminopimelate decarboxylase, an essential enzyme in bacterial lysine biosynthesis. J. Biol. Chem. 278, 18588–18596 (2003). 12637582 10.1074/jbc.M301549200

[b7] Cummins C. S. & Harris H. The chemical composition of the cell wall in some gram-positive bacteria and its possible value as a taxonomic character. J. Gen. Microbiol. 14, 583–600 (1956). 13346020 10.1099/00221287-14-3-583

[b8] Blickling S. *et al.* Reaction mechanism of Escherichia coli dihydrodipicolinate synthase investigated by X-ray crystallography and NMR spectroscopy. Biochemistry 36, 24–33 (1997). 8993314 10.1021/bi962272d

[b9] Paiva A. M. *et al.* Inhibitors of dihydrodipicolinate reductase, a key enzyme of the diaminopimelate pathway of Mycobacterium tuberculosis. Biochim. Biophys. Acta 1545, 67–77 (2001). 11342032 10.1016/s0167-4838(00)00262-4

[b10] Cox R. J., Sutherland A. & Vederas J. C. Bacterial diaminopimelate metabolism as a target for antibiotic design. Bioorganic Med. Chem. 8, 843–871 (2000). 10.1016/s0968-0896(00)00044-4 10881998

[b11] Kefala G. *et al.* Crystal structure and kinetic study of dihydrodipicolinate synthase from Mycobacterium tuberculosis. Biochem. J. 411, 351–360 (2008). 18062777 10.1042/BJ20071360

[b12] Mirwaldt C., Korndörfer I. & Huber R. The crystal structure of dihydrodipicolinate synthase from Escherichia coli at 2.5 A resolution. J. Mol. Biol. 246, 227–239 (1995). 7853400 10.1006/jmbi.1994.0078

[b13] Blickling S. *et al.* Structure of dihydrodipicolinate synthase of Nicotiana sylvestris reveals novel quaternary structure. J. Mol. Biol. 274, 608–621 (1997). 9417939 10.1006/jmbi.1997.1393

[b14] Pearce F. G., Perugini M. a., McKerchar H. J. & Gerrard J. a. Dihydrodipicolinate synthase from Thermotoga maritima. Biochem. J. 400, 359–366 (2006). 16872276 10.1042/BJ20060771 PMC1652817

[b15] Gorman M. A., Janet M., Michael W. & Matthew A. crystallization communications The purification, crystallization and preliminary X-ray diffraction analysis of dihydrodipicolinate synthase from Clostridium botulinum crystallization communications. 206–208 (2008). 10.1107/S1744309108002819 PMC2374160 18323610

[b16] Rice E. A. *et al.* Characterization and crystal structure of lysine insensitive Corynebacterium glutamicum dihydrodipicolinate synthase (cDHDPS) protein. Arch. Biochem. Biophys. 480, 111–121 (2008). 18930704 10.1016/j.abb.2008.09.018

[b17] Blagova E. *et al.* Crystal structure of dihydrodipicolinate synthase (BA3935) from Bacillus anthracis at 1.94 A resolution. Proteins 62, 297–301 (2006). 16287120 10.1002/prot.20684

[b18] Girish T. S., Sharma E. & Gopal B. Structural and functional characterization of Staphylococcus aureus dihydrodipicolinate synthase. FEBS Lett. 582, 2923–2930 (2008). 18671976 10.1016/j.febslet.2008.07.035

[b19] Lawrence M. C. *et al.* Structure and mechanism of a sub-family of enzymes related to N-acetylneuraminate lyase. J. Mol. Biol. 266, 381–399 (1997). 9047371 10.1006/jmbi.1996.0769

[b20] Garg A., Tewari R. & Raghava G. P. S. Virtual Screening of potential drug-like inhibitors against Lysine/DAP pathway of Mycobacterium tuberculosis. BMC Bioinformatics 11 Suppl 1, S53 (2010). 20122228 10.1186/1471-2105-11-S1-S53 PMC3009526

[b21] Karsten W. E. Dihydrodipicolinate synthase from Escherichia coli: pH dependent changes in the kinetic mechanism and kinetic mechanism of allosteric inhibition by L-lysine. Biochemistry 36, 1730–1739 (1997). 9048556 10.1021/bi962264x

[b22] Dobson R. C. J. *et al.* Role of arginine 138 in the catalysis and regulation of Escherichia coli dihydrodipicolinate synthase. Biochemistry 44, 13007–13013 (2005). 16185069 10.1021/bi051281w

[b23] Dobson R. C. J., Valegård K. & Gerrard J. A. The crystal structure of three site-directed mutants of Escherichia coli dihydrodipicolinate synthase: further evidence for a catalytic triad. J. Mol. Biol. 338, 329–339 (2004). 15066435 10.1016/j.jmb.2004.02.060

[b24] Pavelka M. S. & Jacobs W. R. Biosynthesis of diaminopimelate, the precursor of lysine and a component of peptidoglycan, is an essential function of Mycobacterium smegmatis. J. Bacteriol. 178, 6496–6507 (1996). 8932306 10.1128/jb.178.22.6496-6507.1996 PMC178536

[b25] Wheeler P. R. & Blanchard J. S. Tuberculosis and the Tubercle Bacillus. American Society of Microbiology (2005).

[b26] Griffin J. E. *et al.* High-resolution phenotypic profiling defines genes essential for mycobacterial growth and cholesterol catabolism. PLoS Pathog. 7, e1002251 (2011). 21980284 10.1371/journal.ppat.1002251 PMC3182942

[b27] Sassetti C. M., Boyd D. H. & Rubin E. J. Comprehensive identification of conditionally essential genes in mycobacteria. Proc. Natl. Acad. Sci. USA. 98, 12712–12717 (2001). 11606763 10.1073/pnas.231275498 PMC60119

[b28] Sassetti C. M., Boyd D. H. & Rubin E. J. Genes required for mycobacterial growth defined by high density mutagenesis. Mol. Microbiol. 48, 77–84 (2003). 12657046 10.1046/j.1365-2958.2003.03425.x

[b29] Puniya B. L., Kulshreshtha D., Verma S. P., Kumar S. & Ramachandran S. Integrated gene co-expression network analysis in the growth phase of Mycobacterium tuberculosis reveals new potential drug targets. Mol. Biosyst. 9, 2798–2815 (2013). 24056838 10.1039/c3mb70278b

[b30] Couper L., McKendrick J. E. & Robins D. J. Pyridine and piperidine derivatives as inhibitors of dihydrdipicolinic acid synthase, a key enzyme in the diaminopimelate pathway to L-lysine. Bioorg. Med. Chem. Lett. 4, 2267–2272 (1994).

[b31] Boughton B. a., Dobson R. C. J., Gerrard J. a. & Hutton C. a. Conformationally constrained diketopimelic acid analogues as inhibitors of dihydrodipicolinate synthase. Bioorganic Med. Chem. Lett. 18, 460–463 (2008). 10.1016/j.bmcl.2007.11.108 18077163

[b32] Turner J. J., Healy J. P., Dobson R. C. J., Gerrard J. a. & Hutton C. a. Two new irreversible inhibitors of dihydrodipicolinate synthase: Diethyl (E,E)-4-oxo-2,5-heptadienedioate and diethyl (E)-4-oxo-2-heptenedioate. Bioorganic Med. Chem. Lett. 15, 995–998 (2005). 10.1016/j.bmcl.2004.12.043 15686899

[b33] Trott O. & Olson A. J. AutoDock Vina: Improving the speed and accuracy of docking with a new scoring function, efficient optimization, and multithreading. J. Comput. Chem. 31, 455–461 (2010). 19499576 10.1002/jcc.21334 PMC3041641

[b34] Kanabus Annabel “Information about Tuberculosis”, GHE, 2016, www.tbfacts.org.

[b35] Towbin H., Staehelin T., Gordon J. & Ross J. Electrophoretic transfer of proteins from polyacrylamide gels to nitrocellulose sheets: procedure and some applications. Proc. Natl. Acad. Sci. USA 59, 423–450 (1995). 10.1073/pnas.76.9.4350 PMC411572 388439

[b36] Perkins D. N., Pappin D. J., Creasy D. M. & Cottrell J. S. Probability-based protein identification by searching sequence databases using mass spectrometry data. Electrophoresis 20, 3551–3567 (1999). 10612281 10.1002/(SICI)1522-2683(19991201)20:18<3551::AID-ELPS3551>3.0.CO;2-2

[b37] Roberts S. J., Morris J. C., Dobson R. C. & Gerrard J. A. The preparation of (S)-Aspartate semi-aldehyde appropriate for use in biochemical studies. Bioorg. Med. Chem. Lett. 13, 265–267 (2003). 12482436 10.1016/s0960-894x(02)00923-x

[b38] Battye T. G. G., Kontogiannis L., Johnson O., Powell H. R. & Leslie A. G. W. iMOSFLM: A new graphical interface for diffraction-image processing with MOSFLM. Acta Crystallogr. Sect. D Biol. Crystallogr. 67, 271–281 (2011). 21460445 10.1107/S0907444910048675 PMC3069742

[b39] Winn M. D. *et al.* Overview of the CCP4 suite and current developments. Acta Crystallogr. Sect. D Biol. Crystallogr. 67, 235–242 (2011). 21460441 10.1107/S0907444910045749 PMC3069738

[b40] McCoy A. J. *et al.* Phaser crystallographic software. J. Appl. Crystallogr. 40, 658–674 (2007). 19461840 10.1107/S0021889807021206 PMC2483472

[b41] Emsley P. & Cowtan K. Coot: Model-building tools for molecular graphics. Acta Crystallogr. Sect. D Biol. Crystallogr. 60, 2126–2132 (2004). 15572765 10.1107/S0907444904019158

[b42] Murshudov G. N. *et al.* REFMAC5 for the refinement of macromolecular crystal structures. Acta Crystallogr. Sect. D Biol. Crystallogr. 67, 355–367 (2011). 21460454 10.1107/S0907444911001314 PMC3069751

[b43] Niesen F. H., Berglund H. & Vedadi M. The use of differential scanning fluorimetry to detect ligand interactions that promote protein stability. Nat. Protoc. 2, 2212–2221 (2007). 17853878 10.1038/nprot.2007.321

[b44] Krishna S. N. *et al.* A fluorescence-based thermal shift assay identifies inhibitors of mitogen activated protein kinase kinase 4. PLoS One 8, 1–10 (2013). 10.1371/journal.pone.0081504 PMC3855329 24339940

[b45] Van Der Spoel D. *et al.* GROMACS: Fast, flexible, and free. J. Comput. Chem. 26, 1701–1718 (2005). 16211538 10.1002/jcc.20291

[b46] Hess B., Kutzner C., van der Spoel D. & Lindahl E. GROMACS 4: Algorithms for Highly Efficient, Load-Balanced, and Scalable Molecular Simulation. J. Chem. Theory Comput. 4, 435–447 (2008). 26620784 10.1021/ct700301q

[b47] Schüttelkopf A. W. & Van Aalten D. M. F. PRODRG: A tool for high-throughput crystallography of protein-ligand complexes. Acta Crystallogr. Sect. D Biol. Crystallogr. 60, 1355–1363 (2004). 15272157 10.1107/S0907444904011679

[b48] Darden T., Perera L., Li L. & Lee P. New tricks for modelers from the crystallography toolkit: The particle mesh Ewald algorithm and its use in nucleic acid simulations. Structure 7, 55–60 (1999). 10368306 10.1016/s0969-2126(99)80033-1

[b49] Hess B., Bekker H., Berendsen H. J. C. & Fraaije J. G. E. M. LINCS: A linear constraint solver for molecular simulations. J. Comput. Chem. 18, 1463–1472 (1997).

[b50] Berendsen H. J. C., Postma J. P. M., van Gunsteren W. F., DiNola a. & Haak J. R. Molecular dynamics with coupling to an external bath. J. Chem. Phys. 81, 3684–3690 (1984).

