# Supplementary material for: Inhibition of Mycobacterium tuberculosis dihydrodipicolinate synthase by alpha-ketopimelic acid and its other structural analogues
Source: Sci Rep. 2016 Aug 9;6:30827. doi: 10.1038/srep30827 (PMC4977564; doi:10.1038/srep30827)
Supplement: Supplementary Information [file srep30827-s1.pdf]

Title

Inhibition of *Mycobacterium tuberculosis* dihydrodipicolinate synthase by alpha-ketopimelic acid and its other structural analogues

Priyanka Shrivastava[1,2], Vikas Navratna[3], Yumnam Silla[4], Rikeshwer P.Dewangan[1], Atreyi Pramanik[1], Sarika Chaudhary[1], GeethaVani Rayasam[5], Anuradha Kumar[5], Balasubramanian Gopal[3] and Srinivasan Ramachandran[1,2*]

[1]Functional Genomics Unit, Council of Scientific and Industrial Research-Institute of Genomics and Integrative Biology (CSIR-IGIB), South Campus, New Delhi 110 025, India

[2]Academy of Scientific and Innovative Research, CSIR-IGIB South Campus, New Delhi 110025, India

[3]Molecular Biophysics Unit, Indian Institute of Science, Bangalore-560012, India

[4]Biotechnology Group (BIF center), Biological Science & Technology Division (BSTD), CSIR-North-East Institute of Science and Technology (CSIR-NEIST), Jorhat, Assam-785006, India

[5]Open Source Drug Discovery Unit (OSDD), CSIR-IGIB, New Delhi, 110001, India

Corresponding author:

Srinivasan Ramachandran

Room No. 130

CSIR- Institute of Genomics and Integrative Biology

South Campus, Mathura Road

New Delhi 110 025 India

+91-11-29879130, ramu@igib.in, ramu@igib.res.in, ramuigib@gmail.com

Figure S1

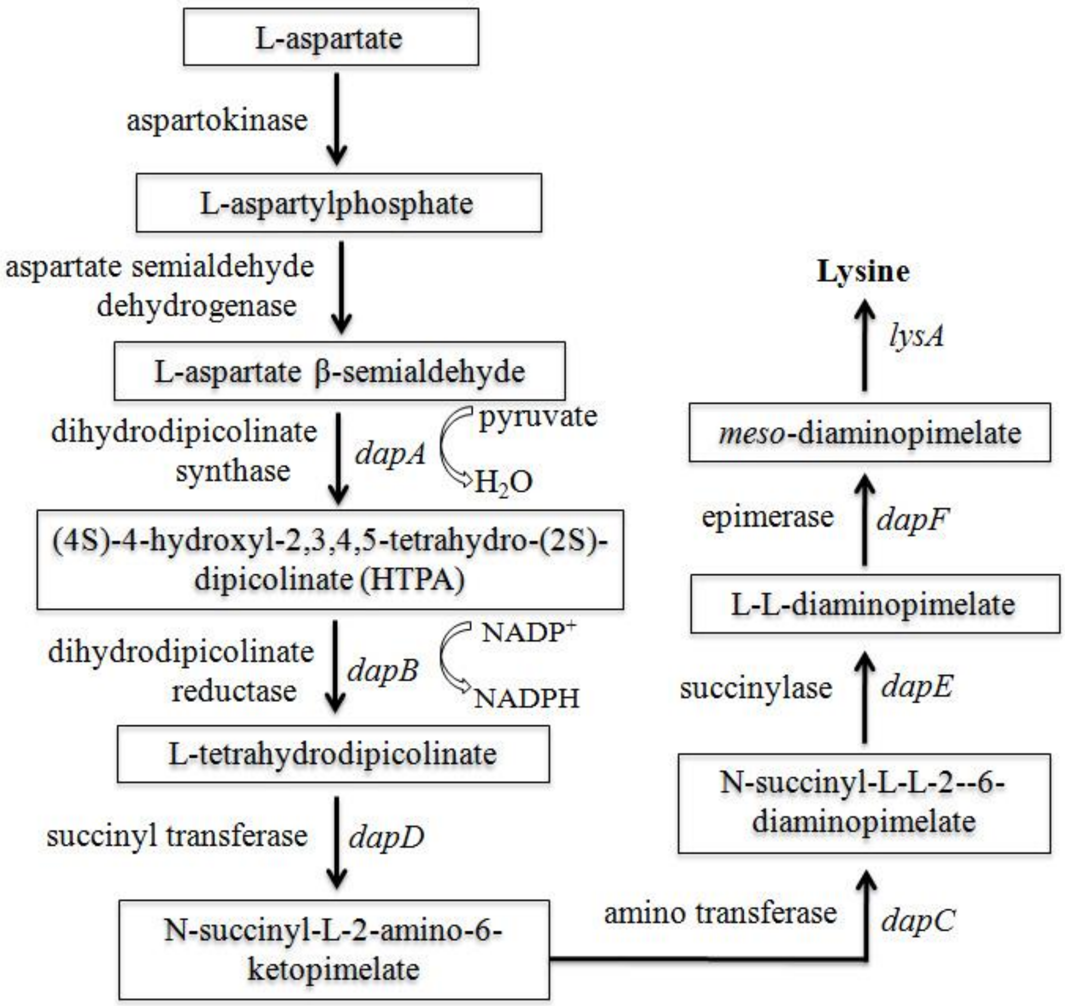

DAP pathway for Lysine biosynthesis:

Dihydrodipicolinate synthase (DapA) catalyzes the first committed step in the pathway, that is the condensation of pyruvate and ASA to form (4S)-4-hydroxyl-2,3,4,5-tetrahydro-(2S)-dipicolinate (HTPA). HTPA is then converted to tetrahydrodipicolinate (THDP) by dihydrodipicolinate reductase (DapB). THDP is subsequently converted in several enzyme-catalyzed steps to meso-diaminopimelate (M-DAP) and lysine.

Figure S2

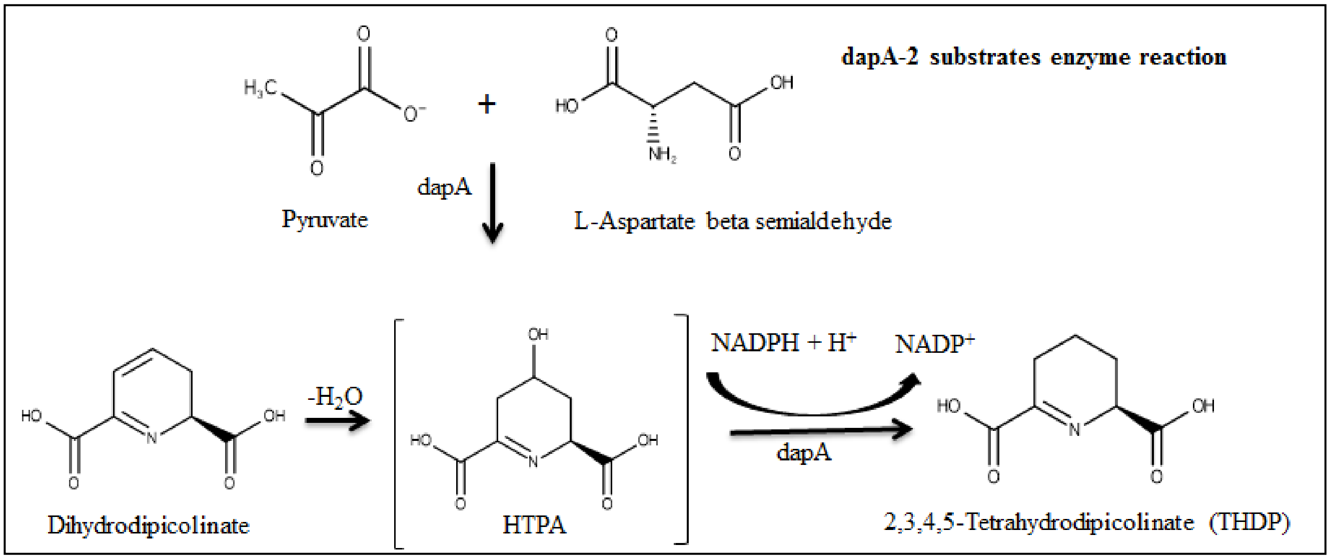

Enzymatic reactions showing THDP formation: The reaction catalyzed by the enzyme Mtb-rdapA in the presence of substrate pyruvate and ASA forms DHDP, which is further reduced to THDP catalysed by the enzyme Mtb-rdapB using NADPH as co-factor.

Figure S3

A

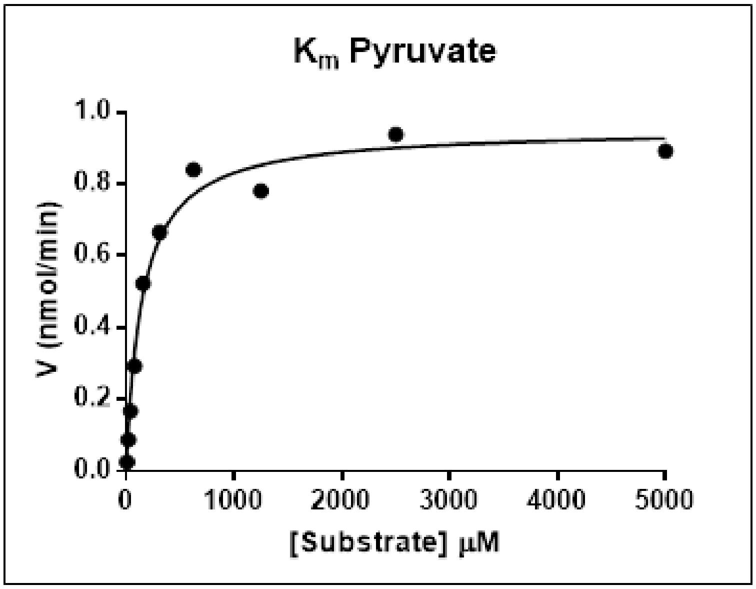

B

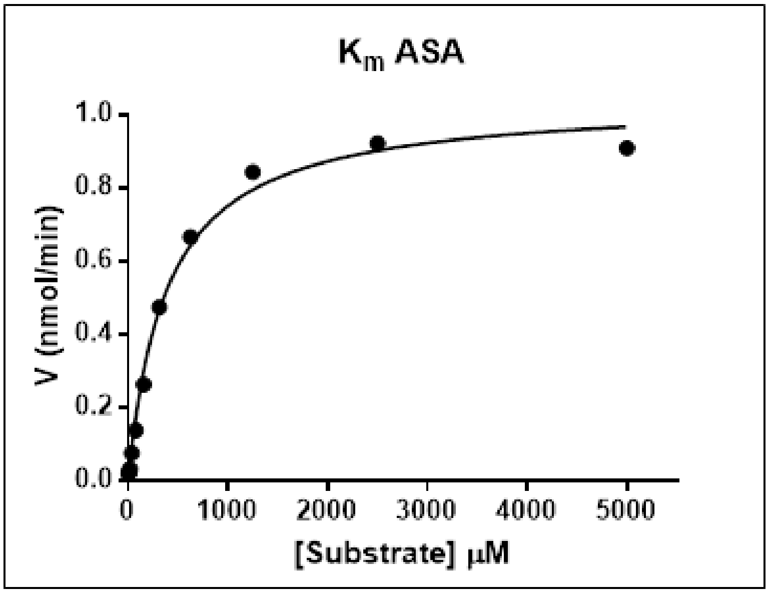

A-B: Kinetic parameter of Mtb-dapA: (A) The K_m for pyruvate with Mtb-dapA was carried out at saturating ASA concentration of 5 mM and (B) for ASA, at saturating pyruvate concentration of 5 mM. The K_m was determined with nonlinear regression analysis showed rate, nanomoles of product (Dihydropicolinate/NADP) formed per unit time, of reaction during 10 minutes was plotted against V_0 (initial substrate concentration) using Michaelis-Menten Plot.

Figure S4 A B

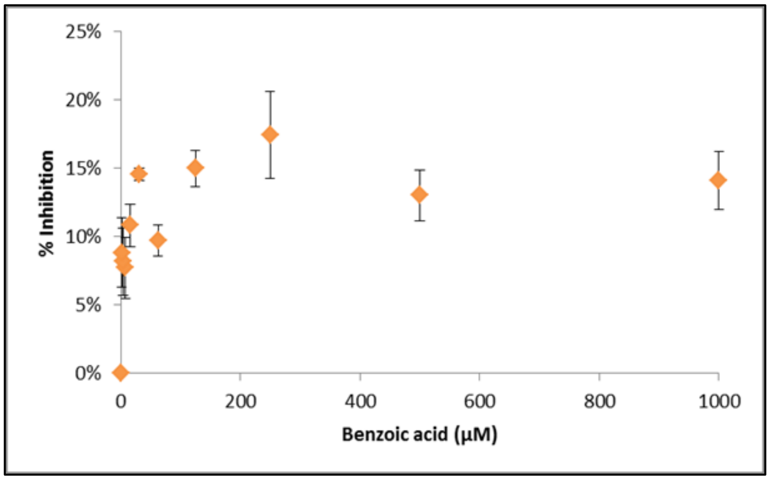

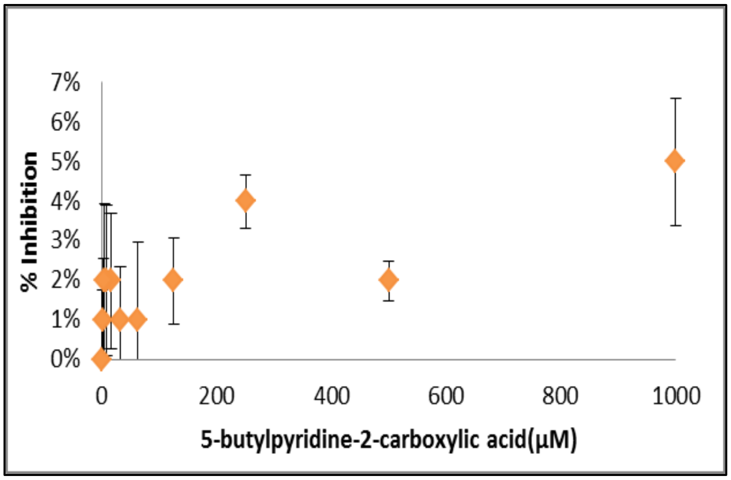

C D

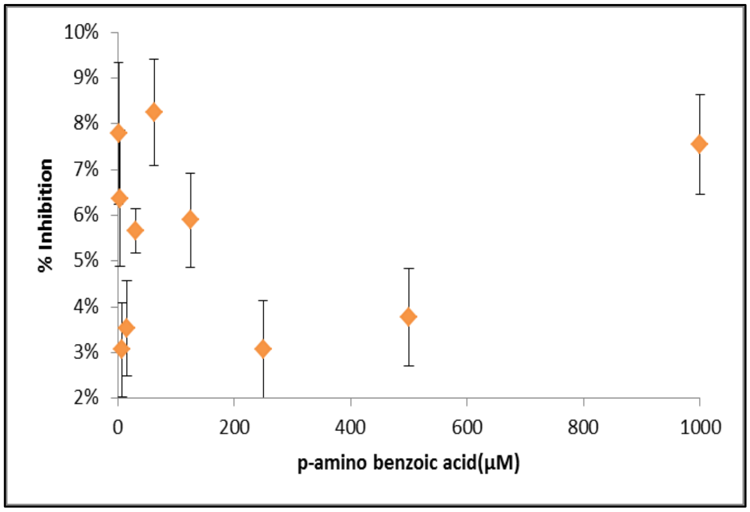

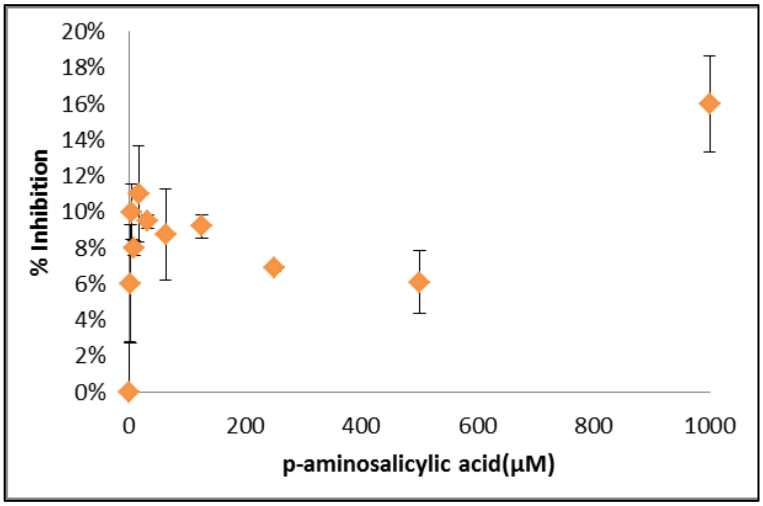

E F

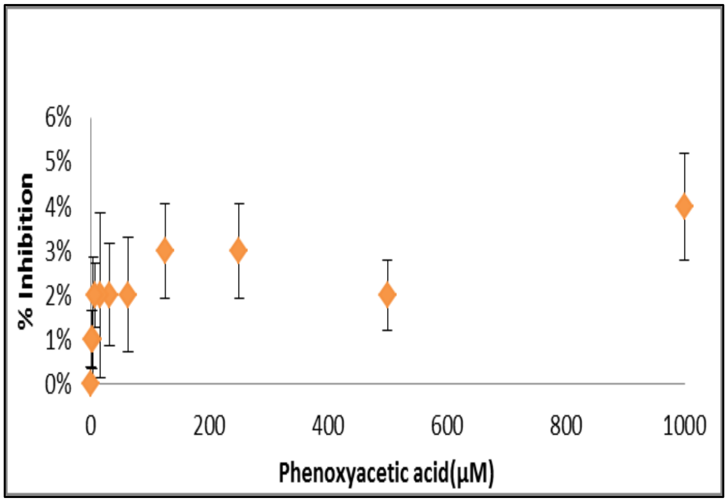

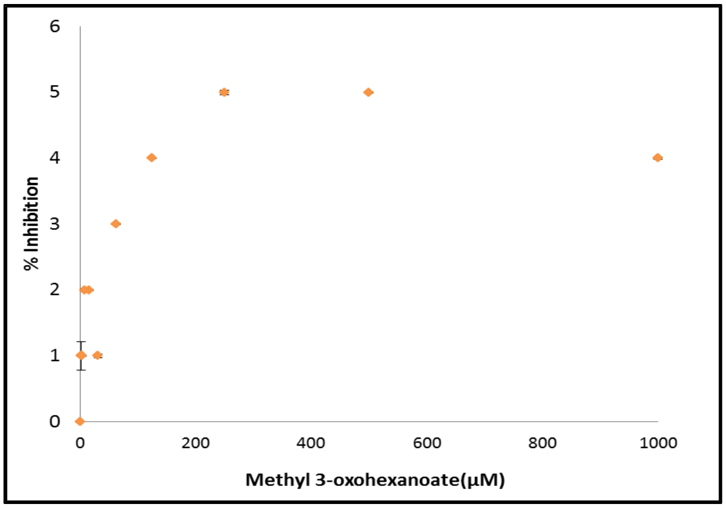

A- F: **IC_{50} analysis of pyruvate structural analogues:** The percentage of Inhibition by each inhibitor relative to control (in absence of inhibitor) was determined using the coupled assay after 5 min pre-incubation of the same across various concentrations of each compound, starting from 0 to 1000 (µM) at conditions of 500µM of pyruvate and 400 µM of ASA in assay mix. Triplicate measurements were taken at each time point and data were plotted with mean ±1SD.

Figure S5

A

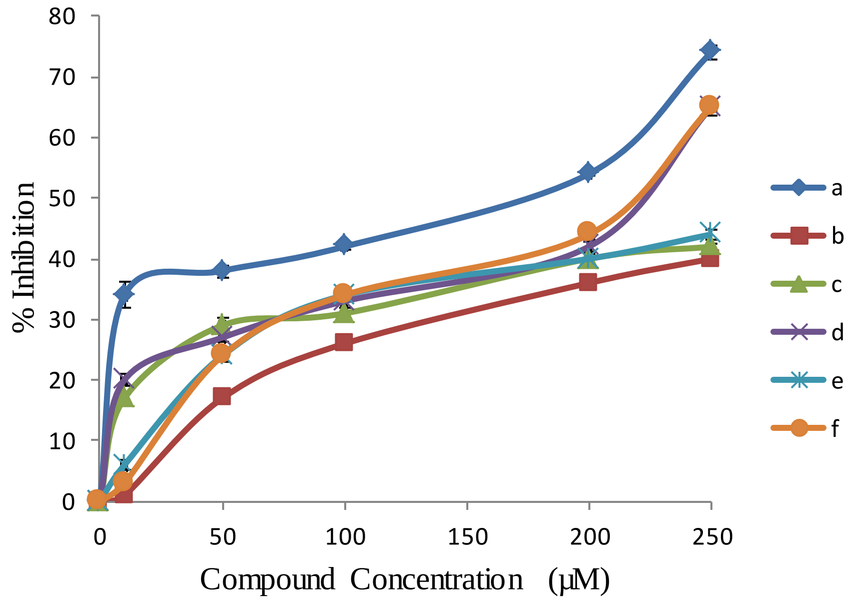

IC50 analysis of α-KPA structural analogues: The percentage of Inhibition by each inhibitor relative to control (in absence of inhibitor) was determined using the coupled assay after 5 min pre-incubation of the same across various concentrations of each compound, starting from 0 to 250 (µM) at conditions of 500µM of pyruvate and 400 µM of ASA in assay mix. Triplicate measurements were taken at each time point and data were plotted with mean ±1SD

a = 2-Hydroxyheptanedioic acid, b = N1-(4-Amino-4-oxobutyl)-N1-methyloxalamide, c = Methyl 4-(2-ethoxy-N-methyl-2-oxoacetamido) butanoate, d = Methyl 5-oxo-5-ureidopentanoate, e = 2-((4-Amino-4-oxobutyl)(methyl) amino)-2-oxoacetic acid, f = 5-(Carbamoylthio) pentanoic acid

B

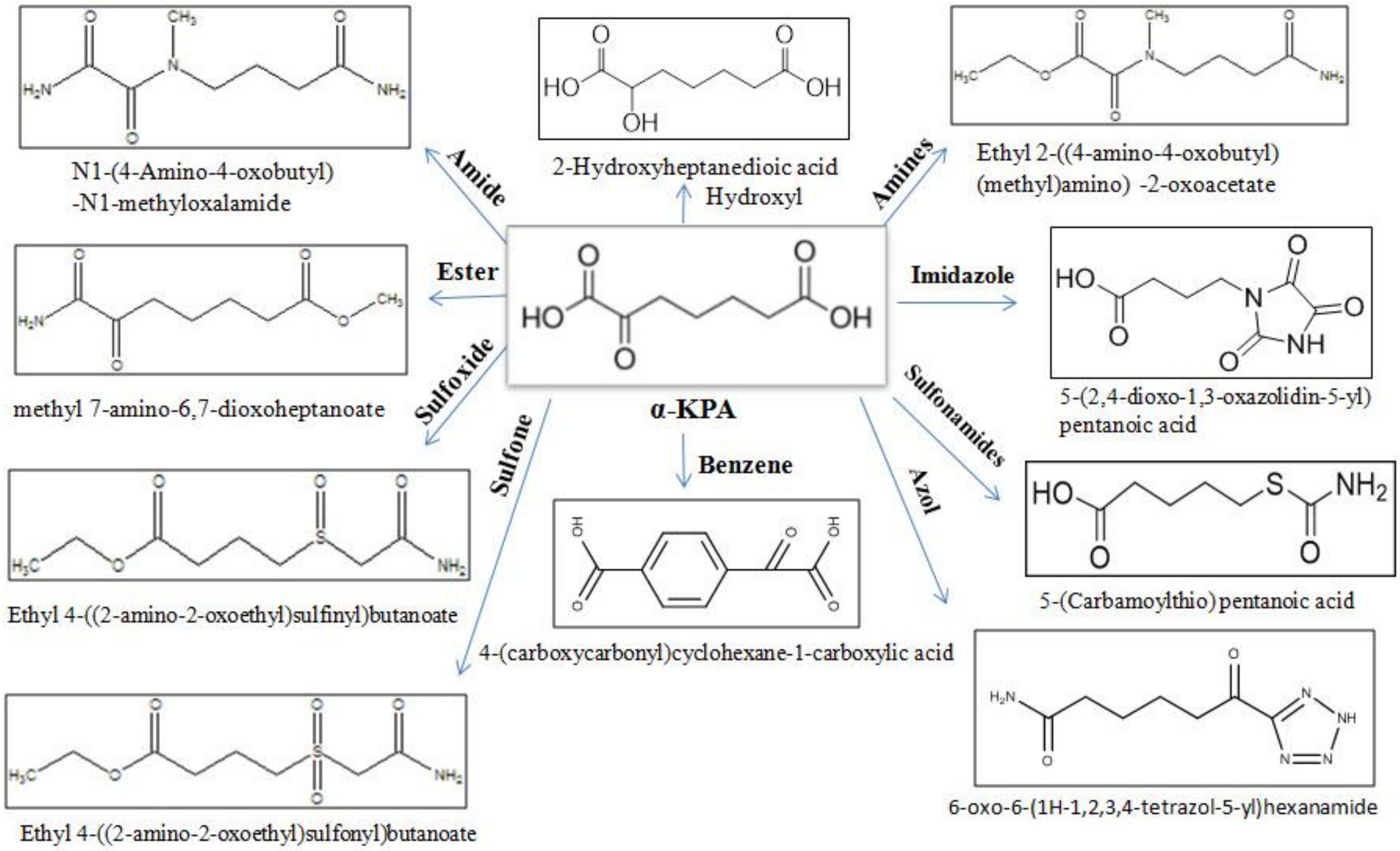

Close analogues of α-KPA: An Intuitive MedChem approach

Design of these inhibitors were based on structural similarity with α-KPA

Figure S6

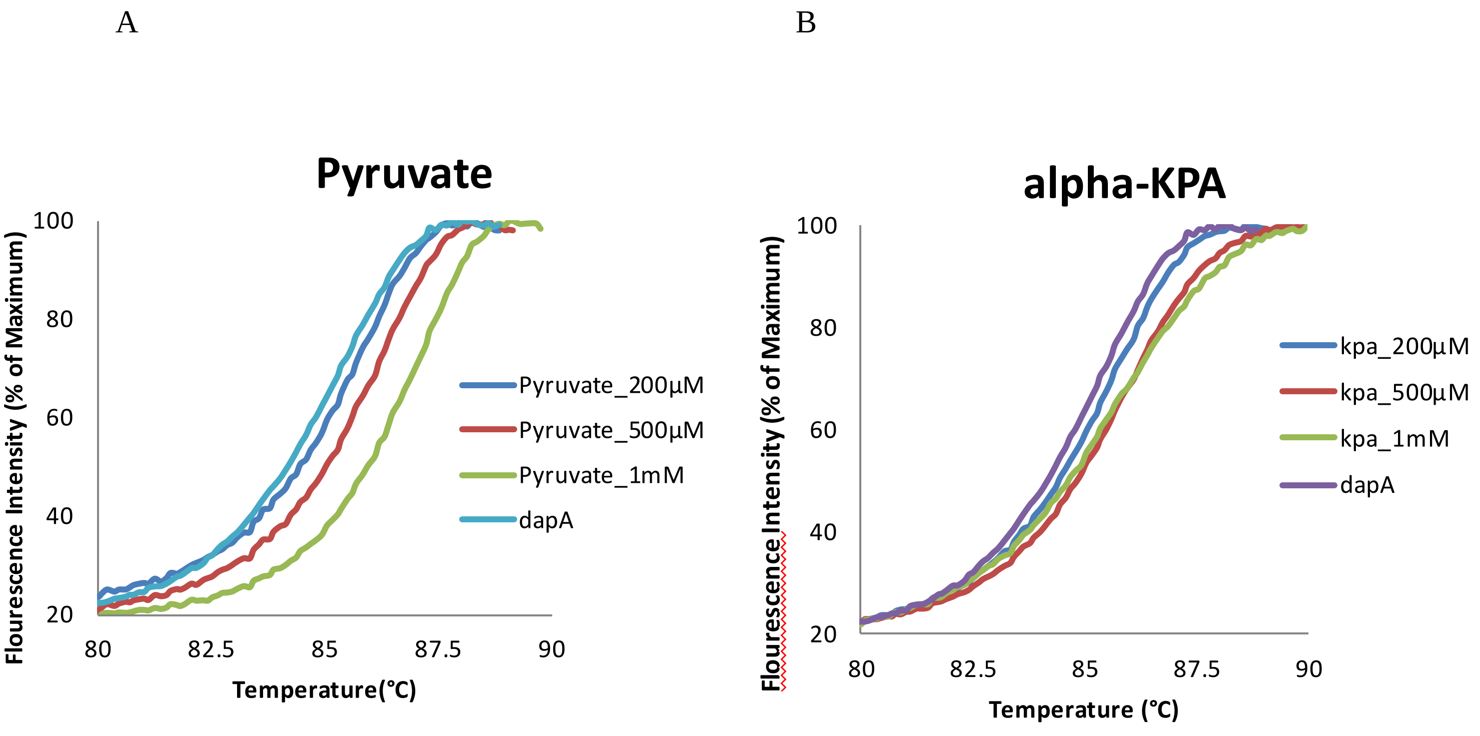

Fluorescence-based thermal shift assay of Mtb-rdapA protein in the presence of either pyruvate or inhibitor α-KPA: (A) Effect of pyruvate in range 0.2mM to 1mM on the denaturation profile of Mtb-rdapA. (B) The effect of α-KPA in the range 0.2mM to 1mM on the denaturation profile of Mtb-rdapA. Experiments were performed in triplicates and the average values are plotted.

Figure S7

MS Daughter Scan of THDP

A

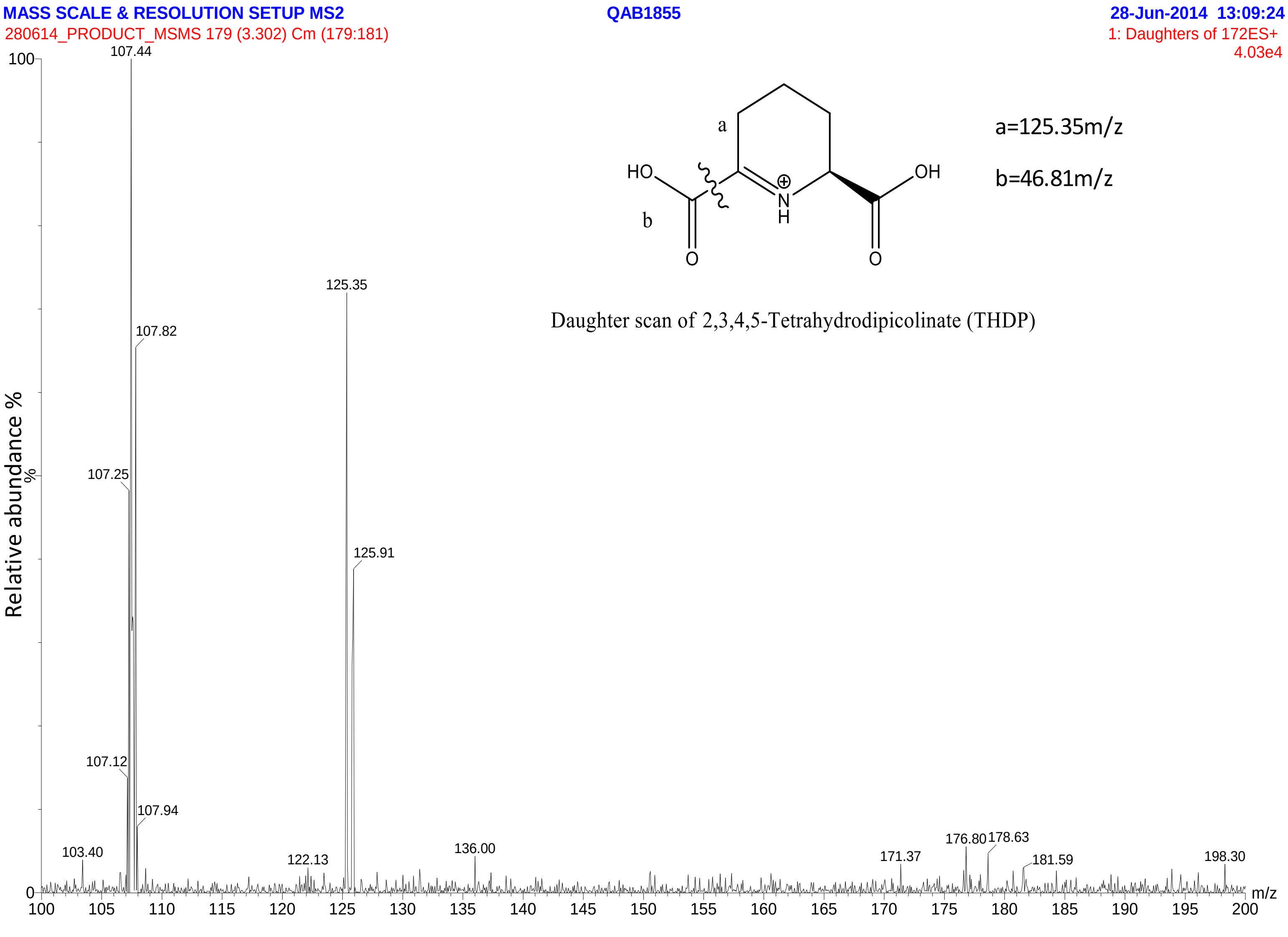

(A) In the MS/MS method, THDP was fragmented into two masses 107.4 and 125.3 by daughter scan showed the presence of parent mass THDP corresponding to product.

B

MS Scan of THDP in the absence of substrate

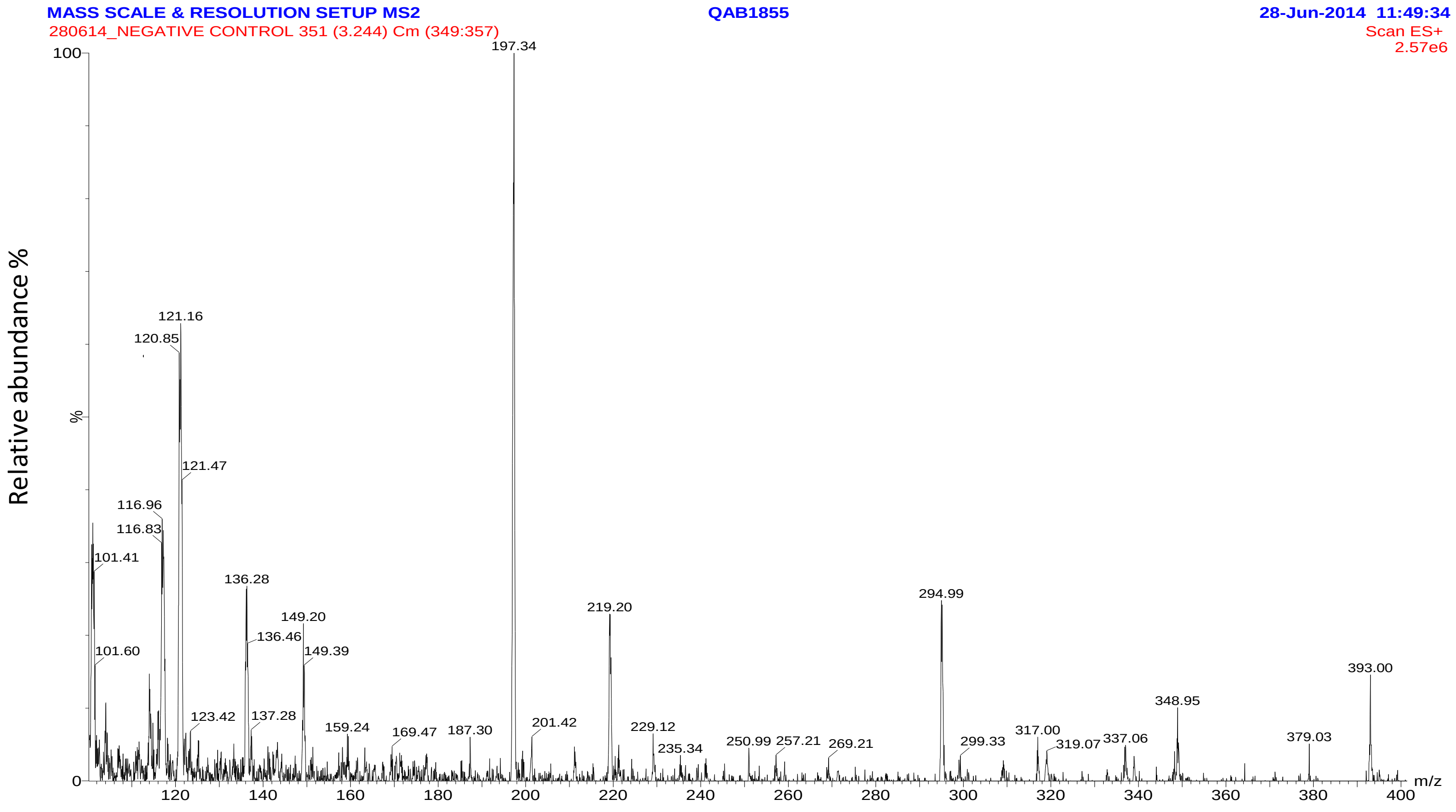

(B) MS analysis of Coupled assay mixture in the absence of ASA. No peak was evident corresponding to THDP mass172.

C

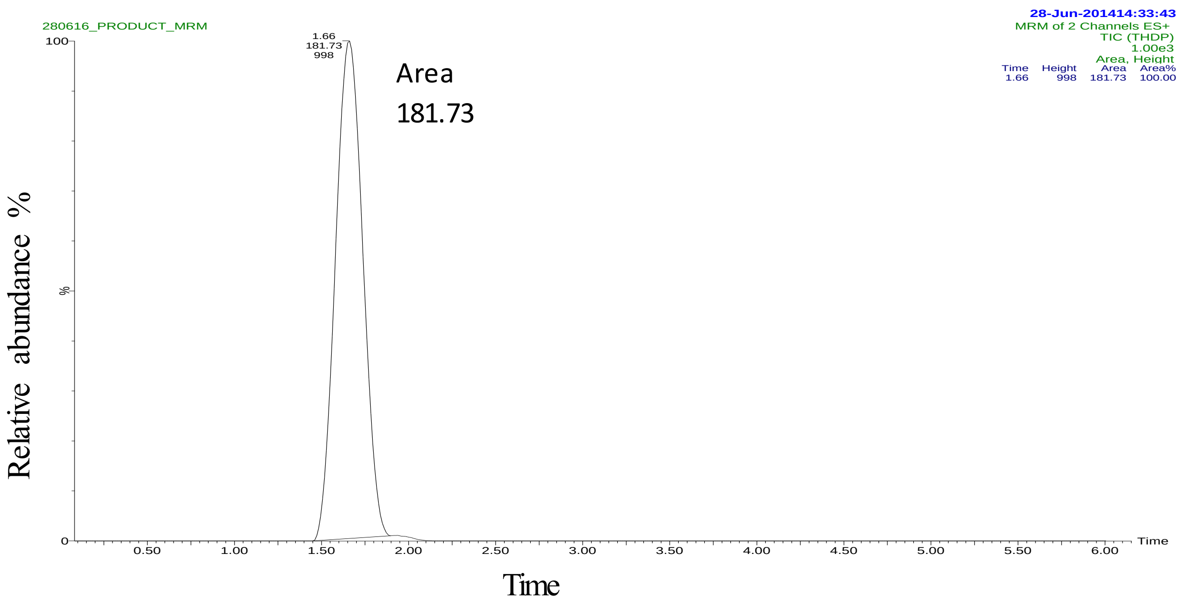

D

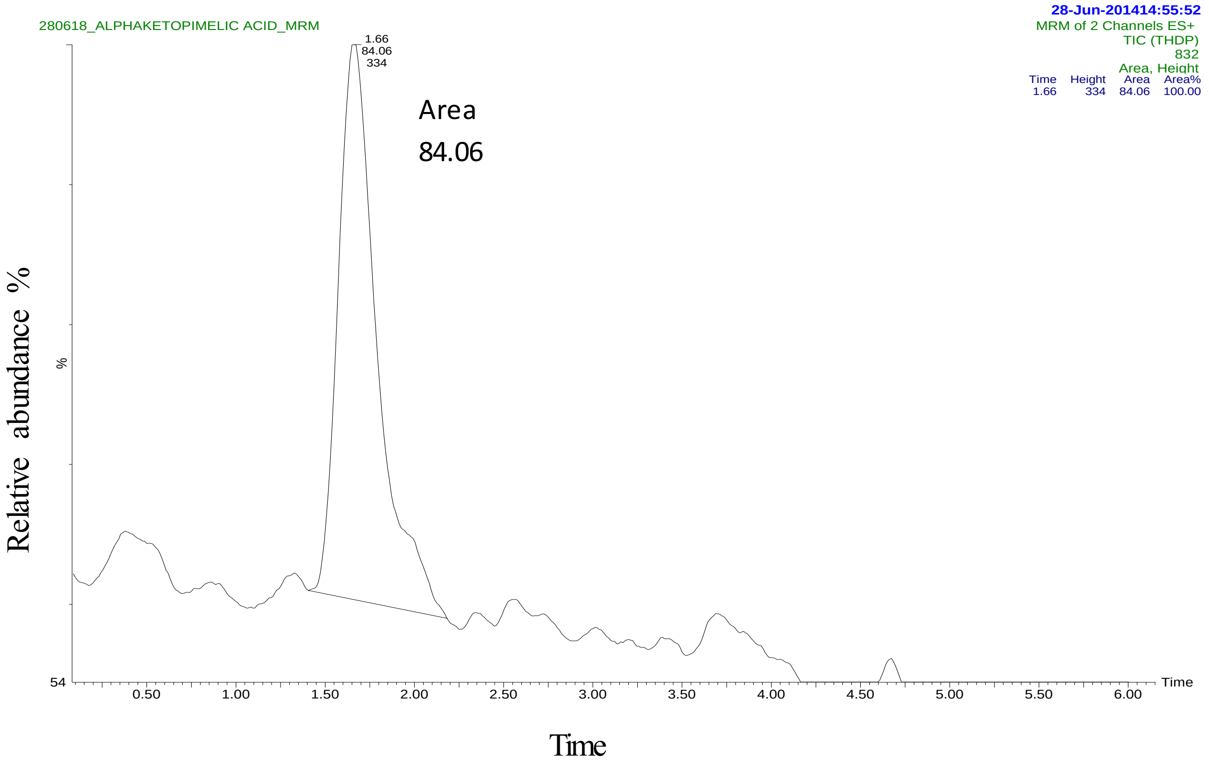

(C) & (D) Multiple reaction monitoring analysis of THDP peak in absence and presence of α-KPA respectively.

Figure S8

A

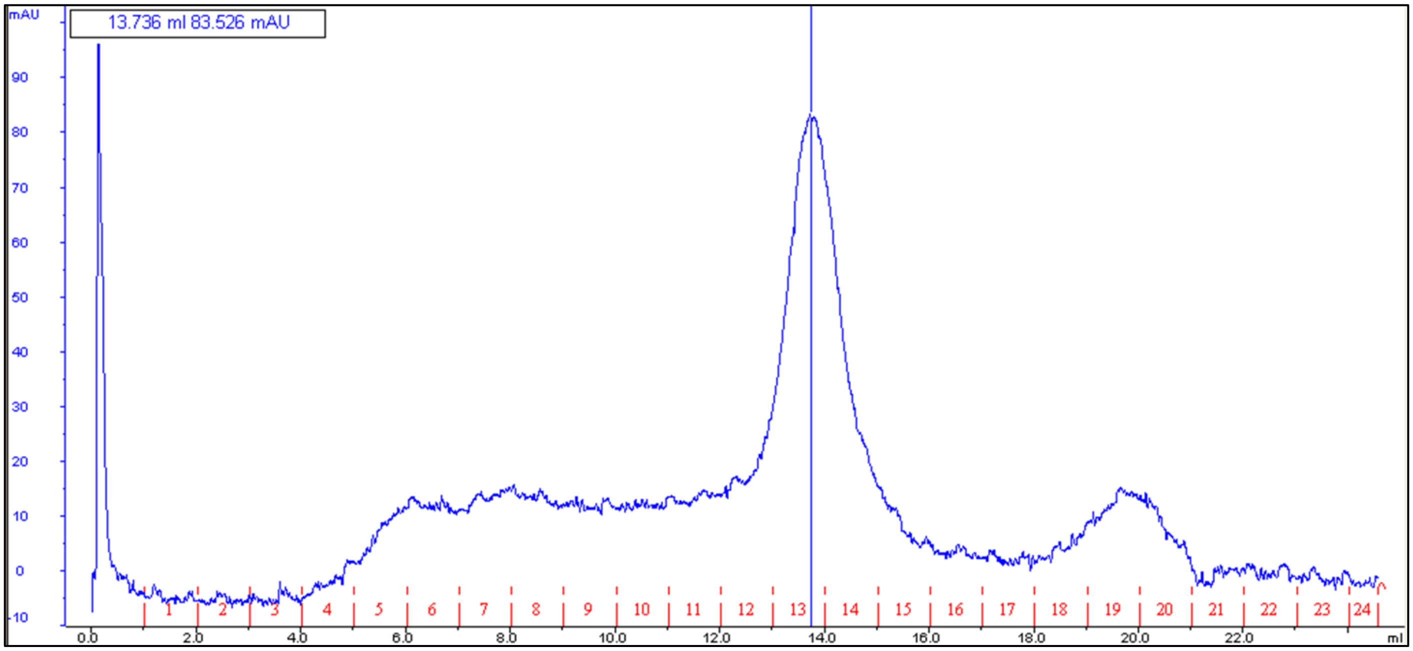

13.736 ml 83.526 mAU
mAU
90
80
70
60
50
40
30
20
10
0
-10
0.0
2.0
4.0
6.0
8.0
10.0
12.0
14.0
16.0
18.0
20.0
22.0
ml
1 2 3 4 5 6 7 8 9 10 11 12 13 14 15 16 17 18 19 20 21 22 23 24

B

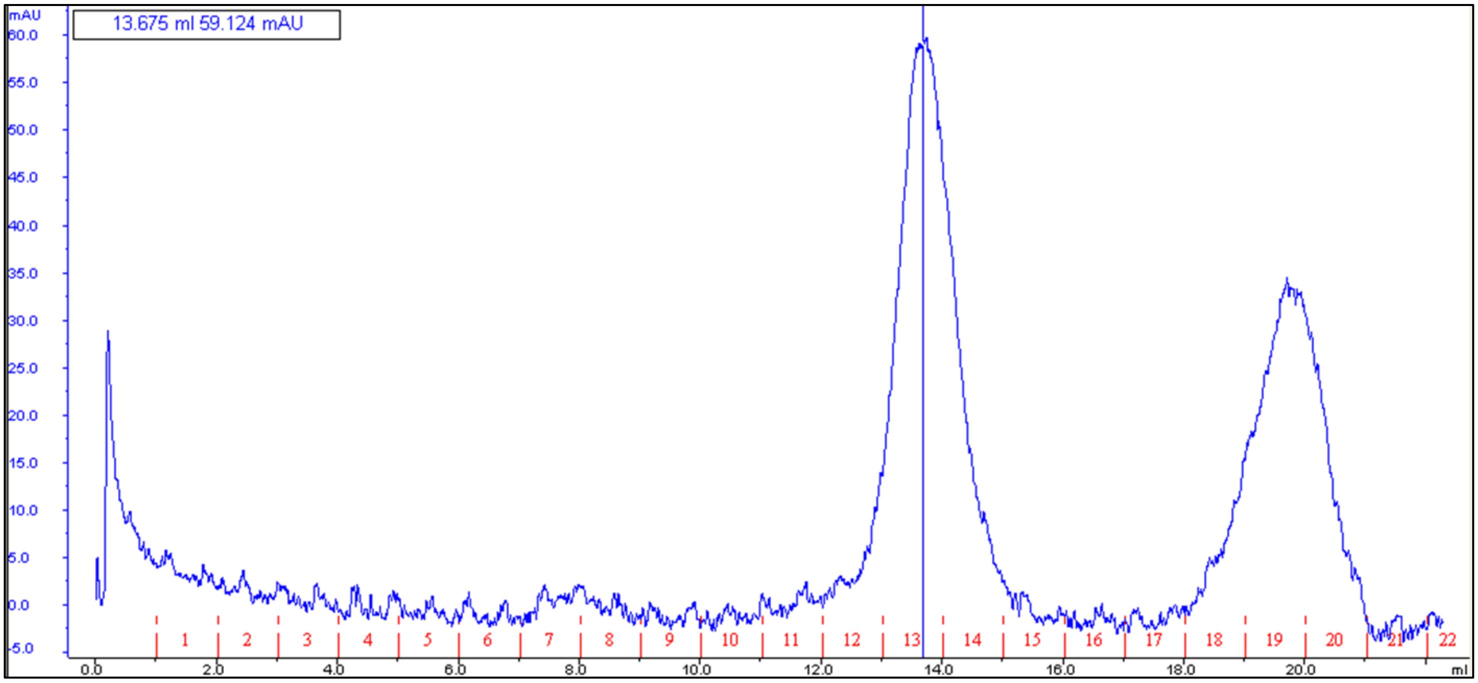

13.675 ml 59.124 mAU
mAU
60.0
55.0
50.0
45.0
40.0
35.0
30.0
25.0
20.0
15.0
10.0
5.0
0.0
-5.0
0.0
2.0
4.0
6.0
8.0
10.0
12.0
14.0
16.0
18.0
20.0
ml
1 2 3 4 5 6 7 8 9 10 11 12 13 14 15 16 17 18 19 20 21 22

Effects of α-KPA towards oligomerizaton state of dapA

Mtb-rdapA incubated with α-KPA at (A) 1:5 and (B) 1:10 ratios at 37°C for 5 min resulted in two peaks one at 13ml elution fraction corresponding to Mtb-rdapA tetramer molecular weight of 144kDa and second one at 18 to 22 ml fraction corresponding to α-KPA.

Figure S9

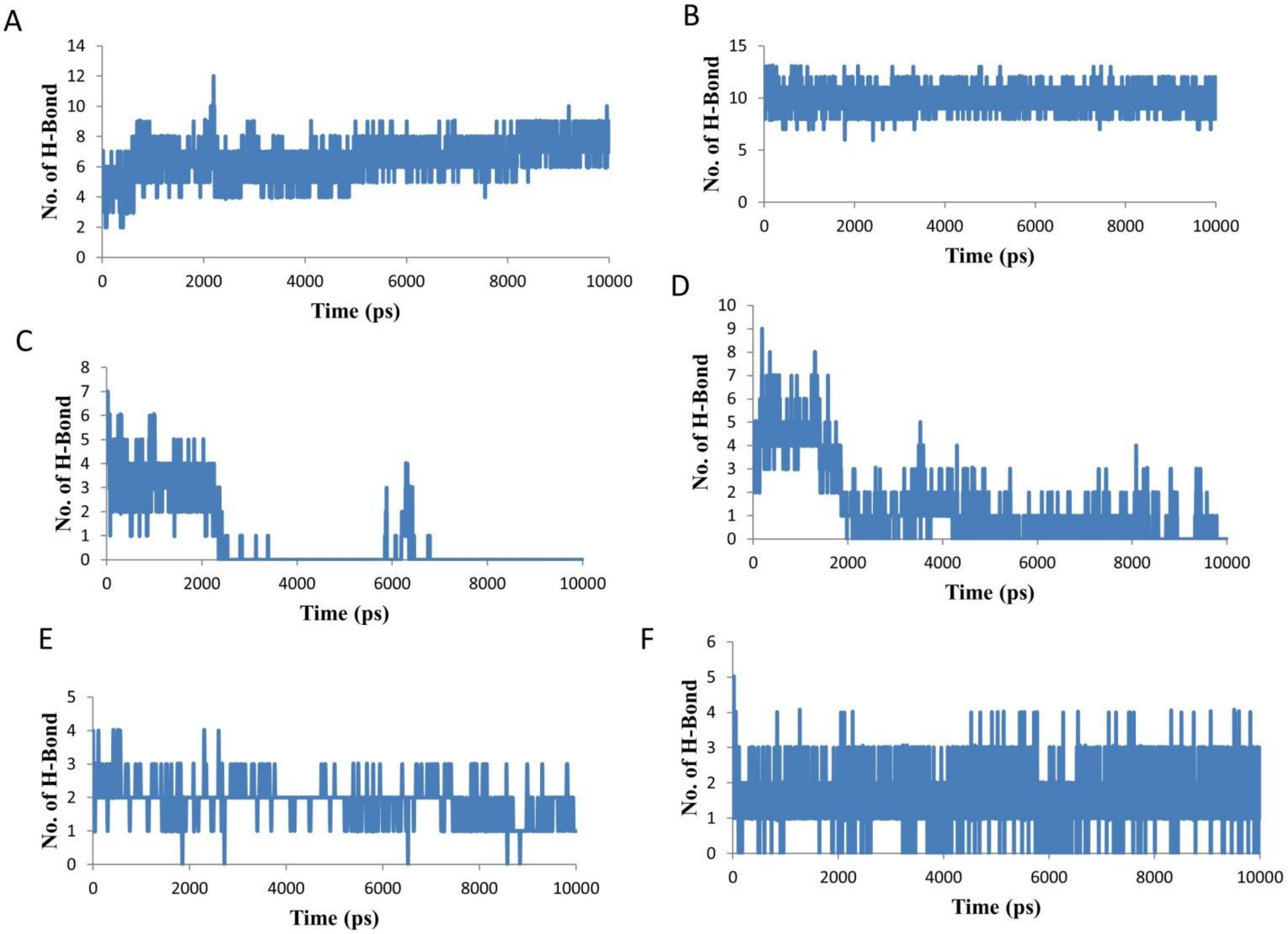

Figures representing the formation of hydrogen bonds between the ligands and Mtb-rDapA during the entire simulation: (A) Methyl 5-oxo-5-ureidopentanoate (B) 2-Hydroxyheptanedioic acid (C) Methyl 4-(2-ethoxy-N-methyl-2-oxoacetamido) butanoate (D) N1-(4-Amino-4-oxobutyl)-N1-methyloxalamide (E) 5-(Carbamoylthio) pentanoic acid and (F) 2-((4-Amino-4-oxobutyl)(methyl) amino)-2-oxoacetic acid with Mtb-rDapA complex is shown.

Figure S10

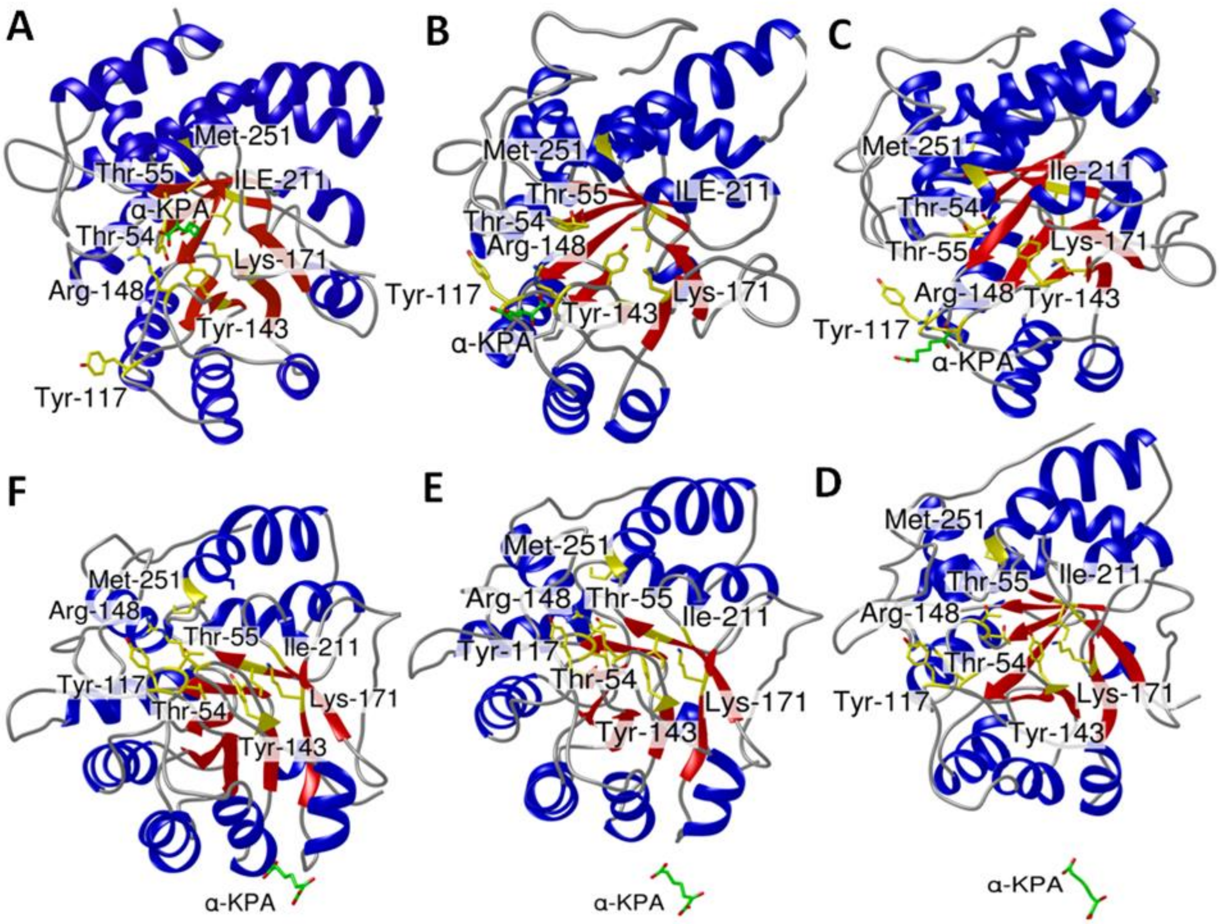

MD trajectory snapshot conformation of α -KPA bound Mtb dapA.

Structures were collected at different time points represented as A-> 1ns frame, B-> 100ns, C-> 200ns, D->300ns, E->400ns, F-> 500ns. The α- helix, β-strand and coil are shown in blue, red and dark gray ribbons. The active site amino acid residues of Mtb dapA and α-KPA are shown as stick in yellow and green respectively. N and O atoms are drawn in blue and red respectively. H-atoms have been removed for clarity and all the active site residues are labeled.

Figure S11

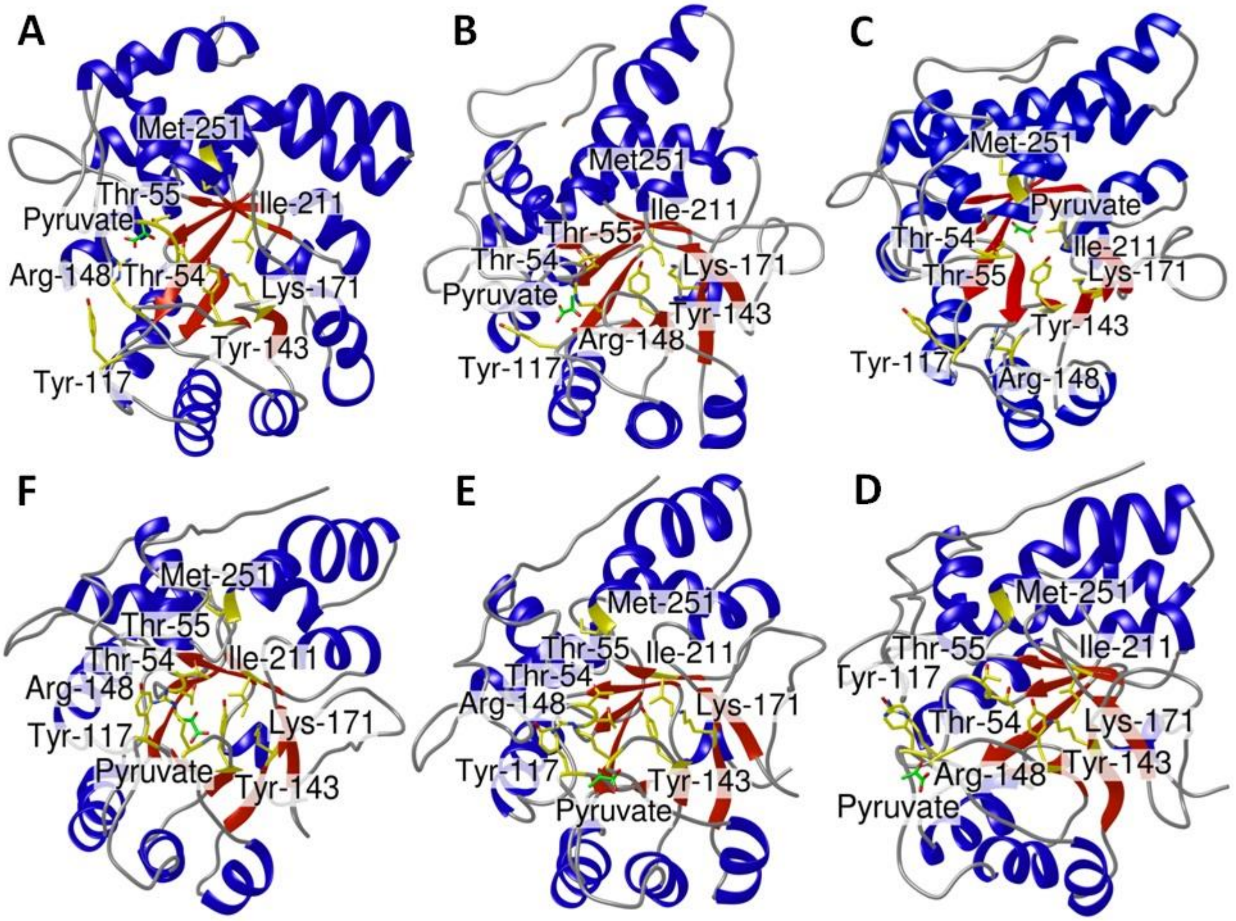

MD trajectory snapshot conformation of Pyruvate bound Mtb dapA taken from different time points (same as in Figure S10).The α- helix, β-strand and coil are represented as blue, red and dark gray ribbons respectively. The active site regions of Mtb dapA and pyruvate are represented as stick in yellow and green respectively. MD Snapshots collected at different time points are represented as A-> 1ns frame, B-> 100ns, C-> 200ns, D->300ns, E->400ns, F-> 500ns.

Figure S12

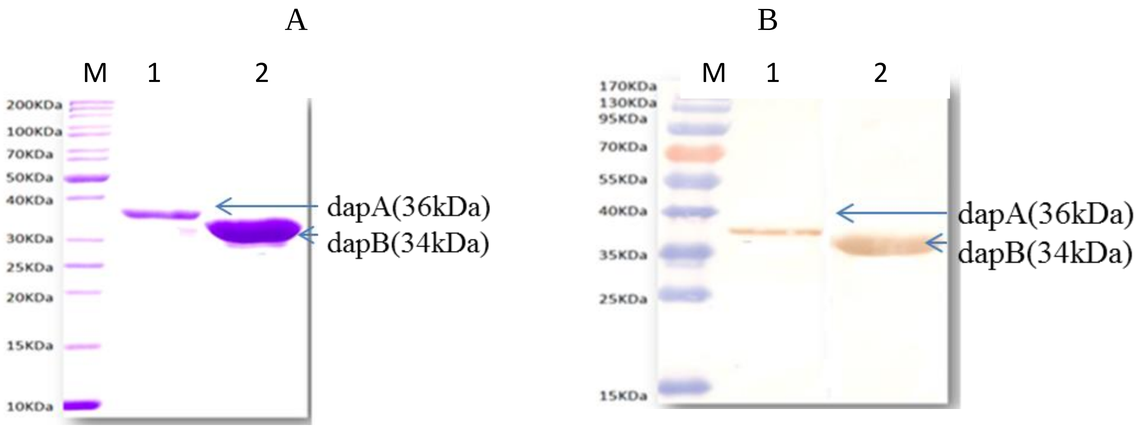

(A) Purified recombinant proteins of Mtb-rdapA and Mtb-rdapB run on a 12% SDS-PAGE and stained with Coomassie Brilliant Blue. Lane M is molecular weight marker (ThermoFisher SCIENTIFIC, USA) and Lane 1 and Lane 2 are purified recombinants Mtb-rdapA and Mtb-rdapB respectively.

(B) Western Blotting with horseradish peroxidase (HRP) conjugated anti-histidine antibodies. Lane M is molecular weight marker (ThermoFisher SCIENTIFIC, USA) and Lane 1 and Lane 2 are the recombinants Mtb-rdapA and Mtb-rdapB respectively.

Figure S13

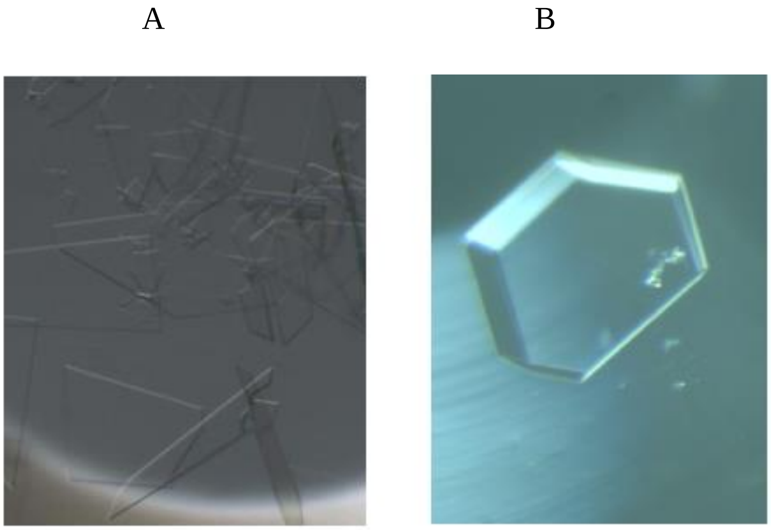

Crystals of the Mtb-rdapA- α-ketopimelic acid complex (A) Preliminary plate like crystals were obtained in the condition containing 1.4M Sodium Citrate Dihydrate, 0.1M HEPES, pH7.5. (B) Crystals obtained in an optimized condition containing 1.34M tri-sodium citrate, 0.1M Tris-HCl, pH7.5 and 1:10 ratio of Mtb-rdapA: α-ketopimelic acid
